# A Case Study on DNN-Based Surface Roughness QA Analysis of Hollow Metal AM Fabricated Parts in a DT-Enabled CW-GTAW Robotic Manufacturing Cell †

**DOI:** 10.3390/s26010004

**Published:** 2025-12-19

**Authors:** João Vítor A. Cabral, Alberto J. Alvares, Antonio Carlos da C. Facciolli, Guilherme C. de Carvalho

**Affiliations:** Department of Mechanical Engineering, University of Brasilia, Campus Darcy Ribeiro, Brasilia 70910-900, DF, Brazil; alvares@alvarestech.com (A.J.A.); gccarval@unb.br (G.C.d.C.)

**Keywords:** additive manufacturing, 3D point clouds, digital twin, neural networks, data integration, interoperability, Industry 4.0, cold wire GTAW

## Abstract

In the context of Industry 4.0, new methods of manufacturing, monitoring, and data generation related to industrial processes have emerged. Over the last decade, a new method of part manufacturing that has been revolutionizing the industry is Additive Manufacturing, which comes in various forms, including the more traditional Fusion Deposition Modeling (FDM) and the more innovative ones, such as Laser Metal Deposition (LMD) and Wire Arc Additive Manufacturing (WAAM). New technologies related to monitoring these processes are also emerging, such as Cyber-Physical Systems (CPSs) or Digital Twins (DTs), which can be used to enable Artificial Intelligence (AI)-powered analysis of generated big data. However, few works have dealt with a comprehensive data analysis, based on Digital Twin systems, to study quality levels of manufactured parts using 3D models. With this background in mind, this current project uses a Digital Twin-enabled dataflow to constitute a basis for a proposed data analysis pipeline. The pipeline consists of analyzing metal AM-manufactured parts’ surface roughness quality levels by the application of a Deep Neural Network (DNN) analytical model and enabling the assessment and tuning of deposition parameters by comparing AM-built models’ 3D representation, obtained by photogrammetry scanning, with the positional data acquired during the deposition process and stored in a cloud database. Stored and analyzed data may be further used to refine the manufacturing of parts, calibration of sensors and refining of the DT model. Also, this work presents a comprehensive study on experiments carried out using the CW-GTAW (Cold Wire Gas Tungsten Arc Welding) process as the means of depositing metal, resulting in hollow parts whose geometries were evaluated by means of both 3D scanned data, obtained via photogrammetry, and positional/deposition process parameters obtained from the Digital Twin architecture pipeline. Finally, an adapted PointNet DNN model was used to evaluate surface roughness quality levels of point clouds into 3 classes (good, fair, and poor), obtaining an overall accuracy of 75.64% on the evaluation of real deposited metal parts.

## 1. Introduction

In light of Industry 4.0, a convergence of technologies such as the Internet of Things (IoT), big data, and Artificial Intelligence (AI) has been driving innovation in industrial applications. In parallel, new manufacturing paradigms have emerged, particularly in Additive Manufacturing (AM), including techniques like Wire Arc Additive Manufacturing (WAAM). In this work, the WAAM process was carried out using Cold Wire Gas Tungsten Arc Welding (CW-GTAW) equipment, which deposits metal along a pre-programmed 3D path derived from slicing the part’s 3D representation.

According to Pramanik et al. [1], key pillars of Industry 4.0 include Cyber-Physical Systems (CPSs), Digital Twins (DTs), big data analytics, and cognitive edge computing, which collectively enable the integration of AI and Machine Learning (ML) into manufacturing analysis. The implementation of these pillars has led to significant advancements such as end-to-end quality and resource control, enhanced Human–Machine Interfaces (HMIs), and optimized decision-making processes [2].

In the context of AM, big data analysis enables a range of applications, including predictive maintenance and real-time or near real-time anomaly detection during fabrication [3]. Another promising application involves leveraging DTs to identify imperfections in manufactured parts by comparing them against predefined quality references derived from CAD models. This work builds upon that principle, which will be elaborated throughout the following sections.

While Digital Twins are increasingly used for monitoring, few works have leveraged them for comprehensive data analysis of manufactured parts using 3D models. To address this gap, this project proposes a novel data analysis pipeline built upon a Digital Twin-enabled dataflow. The pipeline’s core methodology consists of analyzing the surface roughness quality of metal AM-manufactured parts by applying a Deep Neural Network (DNN). This approach enables the assessment and tuning of deposition parameters by directly comparing the final part’s 3D representation—obtained via photogrammetry scanning—with the positional data acquired from the DT cloud database, allowing for the identification and precise localization of defects. This correlation with the history of deposition parameters collected from the DT pipeline directly supports the fine-tuning of input parameters throughout the deposition experiments. This work presents a comprehensive case study using the Cold-Wire Gas Tungsten Arc Welding (CW-GTAW) process to fabricate hollow parts. Specifically, an adapted PointNet DNN model is developed to evaluate and classify 3D point cloud segments into three distinct surface quality classes (good, fair, and poor), providing a new framework for semi-automated quality assurance in AM.

In contrast to existing QA frameworks for metal AM, which predominantly rely on 2D image-based analysis, acoustic signal analysis or thermal monitoring [4] to detect visual defects, this research introduces a distinct geometry-centric methodology. While image-based approaches excel at identifying surface anomalies such as discoloration or spatter, they often overlook the volumetric and topological irregularities that define the structural quality of the deposited bead. The specific novelty of this work lies in establishing a direct analytical link between 3D geometric data and process stability through unsupervised learning. By integrating spectral graph theory (specifically the Fiedler number) to generate ground truth labels for a 3D Deep Learning model, this pipeline provides a scalable, automated framework for assessing meso-scale surface roughness. This distinguishes the proposed approach from traditional contact-based profilometry or purely visual inspection methods, filling a critical gap in Digital Twin-enabled process monitoring.

The remainder of this article is organized as follows. Section 2 presents a literature review covering AI/ML algorithms and recent computer vision techniques for generating and analyzing point cloud models. Section 3 details the methodology proposed to address the problem. Section 4 discusses the experimental setup and presents the results of applying a Deep Neural Network model adapted from PointNet to real-world data. Section 5 explores the limitations of the model and compares it with similar approaches. Finally, Section 6 concludes the paper and outlines future directions for this research.

## 2. Literature Background

The state of the art on which this article is based is composed of a review of recent advancements made towards computer vision enabled by Artificial Intelligence, clustering methods related to point-cloud technologies, and, finally, neural network-based methods of generating 3D object meshes based on point-cloud clusters. Besides the analytical part of the massive batches of data available through DT-enabled solutions, a small review of related articles on DT implementations that can be used as a basis for analytical model developments is presented in Section 2.4. Concepts related to the process performed by the robotic manufacturing cell will be commented on as well.

In order to clarify this work’s contributions to the state of the art of DT-enabled geometrical analysis of AM fabricated parts comparing to recent similar projects, firstly, in the next subsection, the bibliometrics process used to support the research in question will be presented.

### 2.1. Bibliometrics

Using the Scopus database of articles as the basis of the bibliometric process, recent articles published in the last 7 years in the niche of this current work were separated using the following query:

(digital AND twin AND ((quality AND assurance) OR (qa) OR (tolerance) OR analysis) AND (manufactur*)

This keyword entry resulted in 1426 documents (from 2012 to June 2024) whose information was extracted from the Scopus database into a .RIS file to be analyzed using the free VOSviewer software version 1.6.20.

In order to find the most cited documents and the main connections between the most relevant authors in relation to the research niche addressed, a network of connections was created using the VOSviewer software based on the number of citations (discarding documents with less than 3 citations).

**Figure 1:**
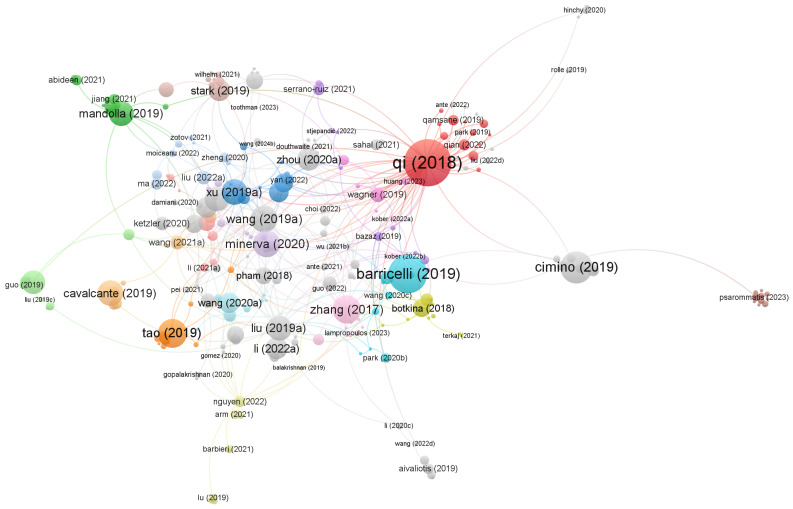
Document cluster link graph. Adjacent clusters have different colors for better visualization; most adherent articles to the current case study are analyzed in Table 1.

This resulted in 658 eligible documents, whose cluster link graph is in the map presented in Figure 1.

Analyzing each cluster and its documents, articles with enough adherence to the theme of this work were selected to be further reviewed. Other review papers were also used to support the research, resulting in more reviewed papers. After the revision process, 10 articles were thoroughly analyzed with their contributions being compared to this current work, resulting in the following Table 1.

Beyond those cited papers, works related to computer vision, AI models, additive manufacturing processes, etc., were also considered as a basis for the methodology of this current work. Those will be further discussed in the following subsections.

### 2.2. ICP Referential Correction of 3D Models

One of the main problems when comparing point clouds or meshes is the reference based on which each point cloud or mesh was obtained. To correct this, the Iterative Closest Point (ICP) algorithm can be applied to the set of points in the generated point clouds. The objective of applying this algorithm to the generated point clouds is to minimize the error in the distances between congruent points when superposing two point-cloud frames, thereby enabling the analysis of errors and tolerances among the manufactured parts. The objective of applying this algorithm to the generated point clouds is to minimize the error in the distances between congruent points when superposing two point-cloud frames, thereby enabling the analysis of errors and tolerances among the manufactured parts.

Newcombe et al. [17] present a method for reconstructing surfaces using Kinect by applying the ICP algorithm to each frame of point cloud data. This approach addresses challenges caused by the sensor’s changing position and angle, allowing for accurate pose estimation and seamless integration of multiple frames into a coherent surface.

Decker et al. [11] propose an enhanced alignment technique for comparing 3D scanned additive manufacturing parts with their original designs. By introducing preprocessing steps—such as manual alignment, segmentation, and resampling—they mitigate common ICP-related errors like misalignment and uneven sampling, ultimately enabling precise deviation analysis for quality assurance.

### 2.3. GTAW Process

The Cold-Wire GTAW process was chosen as the process for depositing metal AM hollow parts in this work, for being a direct energy deposition process that usually results in better superficial finishing and quality when compared to other processes, such as the more commonly used GMAW derivatives [18].

The GTAW process is characterized by the utilization of a non-consumable tungsten electrode, which originates an electric arc by ionizing an inert gas (argon or helium) continuously provided by the GTAW torch during the deposition process. While the torch is moved, a non-heated metal wire is continuously fed to the molten pool under the arc, along the deposition path. The inert gas also protects both the electrode and the molten metal pool against the oxidizing potential of the air, further providing the ionization media [19]. Regarding this process, the main limitation is the orientation of the TCP (Tool Center Point, at the tip of the torch).

In a non-omnidirectional system, variations in feed orientation induce dimensional inconsistencies in the deposited track [20]. To mitigate this, the deposition table is rotated about the *Z*-axis, ensuring that the TCP orientation remains tangent to the tool path. This configuration renders the setup omnidirectional, as the travel direction continuously adjusts to preserve the feed orientation of the filler wire throughout the build. Front feeding is preferred, as it provides superior surface finish, geometric control, and overall quality [21].

As the GTAW WAAM process is characterized by also being a Direct Energy Deposition (DED) process [22], there are 3D part building and layering deposition defects that must be taken into consideration while analyzing manufactured parts and refining deposition parameters. Liu et al. [23] systematically categorize and examine defects in DED, focusing specifically on part quality issues such as geometrical, morphological, and microstructural anomalies. By linking these defects to their root causes and potential solutions, the study aims to equip manufacturers with strategies to mitigate challenges and improve the reliability of metal AM processes. The main defects categorized in the review article are balling (premature liquefaction), dripping (loss of wire–substrate contact), necking (excessive thinning and poor adhesion), overbuilding (excessive layer height), and stubbing (insufficient wire fusion) [24].

### 2.4. Related Works

While the previous subsections addressed the specific algorithmic and physical components of the proposed system, this section reviews integrated Digital Twin architectures and Quality Assurance frameworks. It specifically synthesizes recent state-of-the-art studies and cross-domain methodologies to define the current research gap in geometric-centric process monitoring.

Recently, many applications of DT have been developed with the focus of obtaining huge chunks of data from monitored assets and industrial processes. In this sense, many projects have already applied AI models to analyze data streamed through DT architectures. Regarding the analysis of product and process lifecycles, a comprehensive implementation of a Digital Twin for additive manufacturing is developed by Liu et al. [25]. In their work, many domains of DT applications were considered in the developed framework, such as quality measurement, product design, and process planning, besides the traditional manufacturing and monitoring applications usually found in DT implementations. The extensive cluster variables and parameters used in their pipeline enable a wide range of data analysis; in this regard, a deep learning-enabled defect analysis of deposited layers is also present in the framework.

Charrier et al. [26] address the challenges related to data elaboration and contextualization for the effective supervision of industrial systems. They propose a novel framework and methodology to extend existing DTs by identifying, collecting, and analyzing data from the production process. Their approach uses low-cost IoT sensors to monitor physical processes (such as vibration, acoustic, and current on a CNC lathe) and transforms this data into Key Performance Indicators (KPIs) within the cyber representation, thereby enhancing the DT’s capabilities for real-time performance monitoring.

Doungtap et al. [27] shows the development of a pipeline used for the creation of DT applications based on analyzing 2D images and assets from a database. The data collected is then processed by a pre-trained deep learning model to create a 3D point cloud representation, which will then be post-processed to create a 3D mesh of the original 2D objects. Those generated 3D meshes are then used in a virtual Digital Twin 3D simulation for visualization and appearance customization.

In the specific application context of point cloud data-driven AM, Tashi et al. [15] present a method of generating CAD files to be manufactured through traditional FDM deposition of layers. The real objects were scanned using a 3D scanner or image processing, but a solution for creating point cloud models from 2D shapes through a parametric approach to point generation is also present.

When considering projects that use 3D point cloud modeling to analyze additively manufactured parts, Rao et al. [28] apply a dimensional quality assurance method based on Spectral Graph Theory (SGT) to analyze parts fabricated through polymer extrusion Fused Filament Fabrication (FFF). The dimensional integrity of the tested parts was evaluated through an SGT-based topological invariant quantifier called Fiedler Number (λ2), which was proved as an efficient dimensional quality estimator throughout the analysis developed.

The Digital Twin-based metal AM deposition data collection pipeline in this current paper is based on a previous project in which a Digital Twin was developed for the metal AM robotic cell [29]. This current work is an evolution of a previous similar project by the authors [30], introducing key methodological advancements and a significantly more detailed experimental scope. While the prior work used simulated 3D models, this article shifts to a specific CW-GTAW case study using real parts, necessitating a new data labeling methodology. This significant methodological improvement involves an unsupervised learning step; “ground truth” labels are generated from four experimentally deposited cylinders by applying K-means clustering to their spectral graph metric (the Fiedler number). Furthermore, the segmentation method was fundamentally changed from a height-based slice to a radial (azimuthal) one, allowing for a more effective analysis of defects across the part’s entire height.

The collection of data occurs based on signals monitored by the ABB ICR5 RobotWare 5.13.0 controller. Those signals are then sent to a local TCP/IP server via Ethernet. This server is embedded into an MQTT adapter on a laptop; all the data streamed from the controller is then structured and transmitted through an MQTT dataflow so that a dashboard and 3D simulation of the mirrored movement of the real assets can be established.

The most important feature related to this current work is the data flow that ends in data published in a cloud Firebase Firestore, allowing the history of data to be accessed and analyzed. In this sense, the data stored in the cloud will be used in this work to generate 3D point clouds of the path traversed by the Tool Center Point (TCP) during the deposition of metal. The main objective is to compare real manufactured parts with the data obtained through the DT monitoring. This new application will be detailed in the following sections.

Finally, this article provides a comprehensive experimental analysis absent from the previous work. It explicitly details the CW-GTAW deposition parameters, including a comparative analysis of “step-by-step” versus “helical” deposition strategies. It leverages the Digital Twin’s captured data—such as graphs of arc current, voltage, and *Z*-axis position—to correlate manufacturing parameters directly with the final surface quality. This detailed transformation pipeline—including the specific radial segmentation, data augmentation, and vectorization into HDF5 format—represents a more mature and specialized implementation of the foundational concept. This new, improved methodological process will be further thoroughly explained in the methodology and results sections.

### 2.5. Quality Assurance Analysis on Metal Additively Manufactured Parts

In order to further support the analysis framework discussed through the methodology in Section 3, a revision on the quality assessment of metal AM-based deposited parts is needed.

According to a recently published review by Jin et al. [31], machine learning has a pivotal role in improving additive manufacturing (AM) overall performance and deposition results. Process parameter optimization enables a better topology on fabricated parts and a more uniform deposited metal bead, potentially without defects. Some of the common defects encountered in metal-deposited parts are metal cracks, porosity and residual internal stresses, which might compromise the mechanical strength of the resulting part [32].

In the context of wire arc additive manufacturing (WAAM), Wu et al. [33] propose a heuristic-based analysis (Box–Behnken Design response surface) to find optimized deposition parameters based on three key parameters: auxiliary gas nozzle angle, gas flow rate, and nozzle-to-substrate distance. Those optimizations led to better morphology, refined grains, and improved properties due to the stirring and cooling effects of the auxiliary gas.

Hou et al. [34] use a convolutional neural network (CNN) approach to detect and classify superficial defects on metal AM deposited parts. Using a residual-attention CNN (RA-CNN) algorithm combined with a support vector machine (SVM), it is possible to extract features from images of manufactured parts and classify their defects into many categories, such as pitted surface, scratches, patches, etc., and also in binary classification (defect or non-defect).

Fountas et al. [35] present a machine learning model based on a virus-evolutionary genetic algorithm (VEGA) for parameter optimization over the selective laser sintering—selective laser melting (SLS-SLM) process for metal deposition. The study explores three experimental cases, each with different objectives: maximizing the density of Ti6Al4V specimens, maximizing both hardness and tensile strength of Ti6Al4V samples, and minimizing surface roughness while maximizing density and hardness of L316 stainless steel powder. It is shown in the results that the VEGA algorithm outperforms other ones, such as Greywolf, Multi-verse, Antlion, and dragonfly algorithms, regarding the optimization of the SLS/SLM processes.

On dimensional accuracy of fused deposition modeling (FDM), Mohamed et al. [36] propose a deep learning neural network framework for optimizing the deposition process. The study suggests a new experimental design technique combining definitive screening design (DSD) and deep learning feedforward artificial neural networks (ANNs) to evaluate and predict the effects of six key operating variables on the dimensional accuracy of FDM-fabricated parts. The approach aims to optimize the FDM process by accurately modeling and analyzing the impact of these variables, leading to improved dimensional accuracy and stability of the manufactured parts.

Lee et al. [4] provide a comprehensive overview of quality monitoring techniques in metal additive manufacturing (AM). The paper highlights the similarities between AM and conventional metal manufacturing processes like welding, emphasizing shared challenges such as layer misalignment, dimensional errors, and residual stress. In metal AM, image data helps detect melt pool characteristics, spatter, and keyhole formation while identifying dimensional errors. Plasma spectrum data enables elemental composition analysis and cladding track measurement, improving spectrum acquisition. Acoustic signal data assesses thermal properties and predicts porosity, contributing to overall process optimization.

Li et al. [37] present a deep learning-based approach for quality monitoring in metal additive manufacturing, leveraging semi-supervised learning to handle low-quality images, taken during the inspection process of metal AM fabricated parts.

When compared to other QA inspection methodologies found in the state of the art of metal AM manufacturing, it is clear that not many of them approach this problem with 3D modelling analysis and AI (deep neural networks, machine learning) applied to such a type of data extraction and structuring. Thus, the research novelty of this paper lies in providing a numerical qualitative approach to quality assessment of GTAW metal manufactured parts, with the proposed approach based on comparisons among 3D models and analytical methods applied to those.

Using data acquisition methods supported by a Digital Twin architecture and CAD/CAM deposition planning, it is also possible to support decision making on the refining of the deposition process itself and to compare expected results with those obtained in the real deposited parts. In the following Section 3 and Section 4, the general methodology of the proposed QA approach to the GTAW process in the context of a DT-enabled robotic cell and the results of the analysis framework implementation will be presented and thoroughly discussed.

## 3. Materials and Methods

To develop a quality assurance model for the manufactured parts from the robotic cell, it is necessary to establish a pipeline architecture for processing the data collected from the history of variables available in the cloud Firebase Firestore database.

The proposed pipeline of data analysis will be based on the principles of the ETL cycle: extraction, transformation and load. With that in mind, data extracted from the implemented Firestore database will be organized in batches to be analyzed and transformed according to programs executed by the DT-enabled AM robotic cell. After the application of AI-driven analysis on the data extracted, the processed data may then be uploaded back to a separate collection on the database to be further used at a later moment.

**Figure 2:**
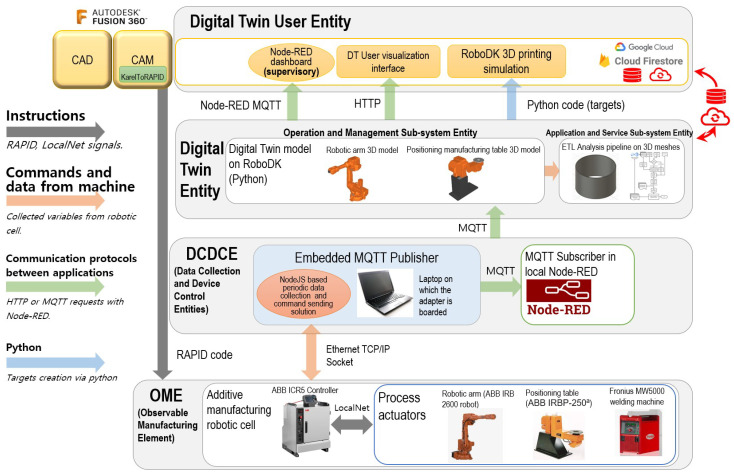
Digital Twin framework architecture based on ISO 23247 applied to a real metal Additive Manufacturing. An ETL pipeline was included as the Application and Service Sub-system Entity (adapted from [29,30]).

A new Digital Twin architecture is conceived based on the previous framework [29], including the proposed ETL pipeline in the Application and Service Sub-system Entity, as presented in Figure 2.

To enable comparisons between expected results and obtained ones along the production cycle, it is necessary to collect positional data for the generation of 3D point-cloud models to be compared with production parameters collected through the Digital Twin. CAD models and meshes of planned 3D parts are also used in this kind of quality assurance analysis.

In the following subsections, the experimental setup and the proposed extraction and transformation of data will be detailed, having in mind the following questions: How can we extract point-cloud data of manufactured parts? How can we align 3D meshes of different representations of the production cycle (point cloud extracted from data collected from the DT and point cloud generated through the finished part)? What methods of surface roughness classification in 3D meshes are suitable for use in the data analysis?

### 3.1. Deposition System Setup

**Figure 3:**
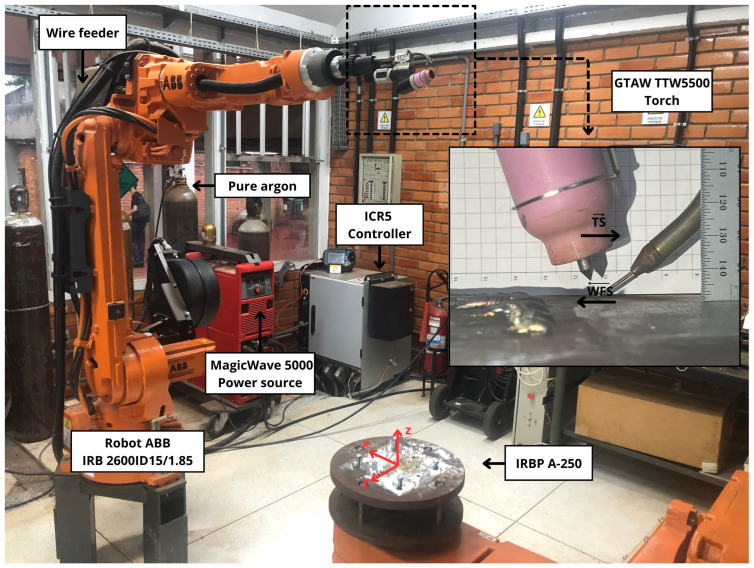
Cold Wire GTAW deposition system. Amplified visualization of the deposition setup is highlighted in the right box, TS: Torch Speed. WFS: Wire Feed Speed.

WAAM was performed with an experimental setup consisting of a 6-axis ABB IRB 2600ID-15/1.8 robotic arm, an IRBP A-250 two-axis positioner, and a Fronius MagicWave 5000 power source, all operated by the IRC5 controller through LocalNet network. Deposition was carried out using a 1.2 mm diameter ER70S-6 mild steel wire as the additive material, onto AISI 1020 steel plates 150 × 150 × 4.75 mm, under pure argon shielding at a 14 L/min flow rate. A fixed arc length of 4 mm was adopted. The configuration of the deposition system is shown in Figure 3.

For the depositions carried out in this work, data from preliminary tests with discontinuous and unidirectional arc planar walls were used. From these tests, TCP positioning data at electrode contact points were collected, enabling the estimation of the average layer height, which was then applied in the programming of the continuous unidirectional deposition path.

Four tubular specimens were produced using different manufacturing strategies, as detailed in Section 4.1. In the first case, the deposition path was the same as that applied in the first, second, and third specimens, but with distinct process parameters. In contrast, the third and fourth specimens were manufactured with identical parameters, differing only in the deposition parameter strategy and the trajectory strategy applied. This experimental design enables a clearer assessment of how parameter variation and deposition path influence the geometry and material distribution of the fabricated cylinders.

### 3.2. Extraction of Data and Methods of Point-Cloud Generation

This section is divided into a few subtopics related to problem solving regarding the extraction and pre-processing of data to be further analyzed. Although positional data is already easily obtained through the cloud database, the raw data must be converted into point-cloud data and must have its density of points corrected to generate 3D meshes afterward. Regarding the 3D CAD models, STL files, which are generally used in 3D printing, can be generated in commonly used programs such as Autodesk Fusion, Rhino, Blender, etc.

The main challenge involved in data extraction is the uniform collection of points for the 3D point-cloud frames, more specifically, the density of the point distribution depending on the sampling method. Moreover, noisy data might also be filtered, generating clusters of scanned objects.

Clustering methods such as DBSCAN clustering [38] may be applied to the generated 3D point cloud in order to filter noisy points generated through program execution and data collection of the Digital Twin pipeline.

After the proper clustering of data points, a very likely problem to be experienced related to the positional data collected via the Digital Twin is the oversampling during the cycles of data collection. Reducing the density of the point-cloud frame is then necessary to diminish the computational power required to generate the 3D meshes; more than that, it is important to have an even density among different frames to facilitate the process of calculating differences.

To correct this problem, a method named Screened Poisson Surface Reconstruction (SPSR) by Kazhdan et al. [39] is used. In this method, the gradient of points along boundaries of surfaces on point-cloud and mesh data is adjusted so a smoother result of the objects’ surfaces is achieved, granting a better distribution of points and better noise filtering.

The second dataset needed for the QA analysis is related to the real manufactured parts. In this case, the SPSR method is also applied because of scanning problems such as insufficient sampling rate, angles of the part not properly covered during the scanning process, and possible duplicate points (the specific implementation of this extraction pipeline is detailed in Section 4.3).

### 3.3. Generation of 3D Meshes

Following the discussion of the generation of 3D models of manufactured parts presented in Section 2.2 and Section 3.2, the next step after correcting the possible errors in the point cloud generation involves generating the 3D meshes based on those positional data clusters.

Since the CAD models used as a base for the manufacturing are already considered a mesh, this process will be skipped on that data. Regarding the positional (x, y, and z axes) data collected via the DT and also the point cloud collected from the manufactured data via photogrammetry, both datasets are suitable for processing through vectorial data using Open3D’s library [40] version 0.9.0, Python version 3.9.2 for creating triangle mesh STL files.

Photogrammetry is based on triangulation, where images taken from two different perspectives help determine the positions of points on an object’s surface. This technique plays a crucial role in depth perception and various spatial analyses. A key challenge in photogrammetry is determining the camera’s intrinsic and extrinsic parameters, along with the spatial coordinates of image points. Intrinsic parameters relate to camera-specific characteristics like focal point and lens distortions, while extrinsic parameters refer to the camera’s position and direction using six parameters: three projection center coordinates (X0, Y0, Z0) and three rotation angles (omega, phi, and kappa) [41].

The implementation of 3D mesh generation algorithms will be further commented on in Section 4 (the visual outputs of the mesh generation process, comparing the scanned geometries against the deposition strategy, are presented in Section 4.1.2).

### 3.4. Deep Neural Network Model for Quality Classification of Manufactured Parts

PointNet [42], a deep neural network designed for processing point cloud data, has shown promising applications in classifying and segmenting 3D models and meshes. Unlike traditional image-based approaches, PointNet directly analyzes 3D point clouds, capturing fine-grained surface details that are crucial for assessing roughness variations. This capability is particularly valuable in GTAW-based AM, where thermal fluctuations and deposition inconsistencies can lead to surface defects.

In a Digital Twin framework, PointNet can be integrated to provide feedback on surface quality, enabling the fine-tuning of deposition parameters. By analyzing point cloud data previously acquired from laser scanners or photogrammetry, the model can detect deviations in roughness all along the surface of the scanned parts, thus enabling data-backed decision making (the architecture of the adapted model is detailed in Section 4.6, and the classification performance on real segments is detailed in Section 4.7).

### 3.5. General Methodology and Pipeline

**Figure 4:**
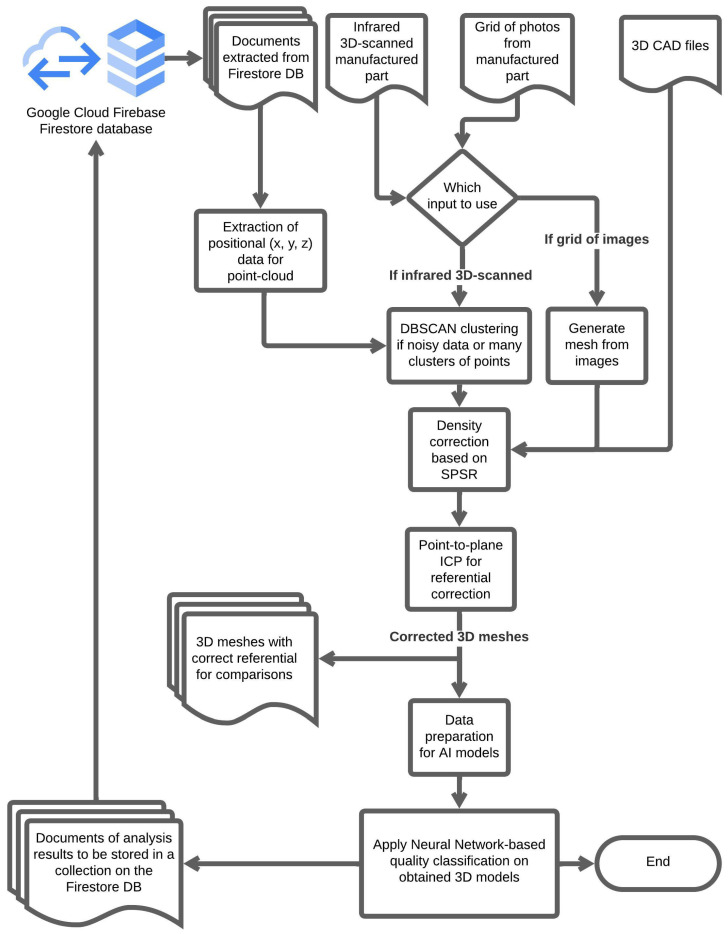
Planned pipeline for data transformation and decision-making analysis based on superficial roughness classification from generated 3D meshes (adapted from [30]).

The methodology presented will be applied to the many generated models of manufactured parts to support decision making and the analysis of the quality of manufactured parts. The pipeline of data makes use of the ETL cycle principles to form a framework for the desired analysis. The general planned pipeline workflow can be seen in Figure 4.

It is important to highlight that, besides the general decision-making analysis applied through the presented pipeline, the generated 3D mesh files might also be used to compare the generated models visually, adding another factor to the machine operators’ decisions regarding the manufacturing process.

In the next section, all the modules and data involved in the pipeline framework illustrated in Figure 4 will be explained in detail with their respective algorithms and results of implementation discussion.

## 4. Results

Throughout this section, the specific parts that compose the entire ETL data pipeline framework proposed in Section 3 will be discussed in its respective implementations and results.

An overview of the parameters utilized on metal deposition at the robotic manufacturing cell will also be provided. This study is aimed at finding the relation between the fine-tuning of the GTAW process and the overall quality of fabricated parts based on the general quality of part classification models.

The proposed quality assurance model is built upon the structure that the framework provides and will be scrutinized in Section 4.6; also, training details and the results of testing with real validation 3D parts with different levels of expected quality will be commented on. Results of the application of said Deep Neural Network model in the context of metal-manufactured part QA analysis are discussed in Section 4.7.

### 4.1. Experimental Methods and Parameters

In this section, the deposition experimental parameters will be discussed. Besides the deposition parameters themselves (arc current, wire feed speed, electrode-to-substrate distance, torch travel angle), there are also parameters related to the robotic arm and positioning table, such as linear speed, deposition base rotation speed, and continuous or discontinuous *Z*-axis ascending rate.

Another aspect of the deposition process to be discussed is the trajectory definition. Two different trajectory approaches were employed. The step-by-step approach consists of a unidirectional trajectory that introduces a 1 mm circumferential shift in the torch ascent points along the cross-section, in order to prevent excessive heat and material accumulation. On the other hand, the helical approach involves a continuous deposition path that reduces the need for arc re-ignition and extinction between layers.

Regarding the deposition strategies, a non-continuous arc was used for the first and second specimens, and a continuous arc for the third and fourth.

All specimens reduced the current as the layer height increased. For instance, the first and second specimens applied a higher reduction of 10 A across the first three layers. The third specimen was produced with a progressive reduction in the current (in decrements of 2 A) without a defined stopping criterion until the final layer. As for the fourth specimen, it followed the same pattern, but the parameters were kept constant from the 22nd layer onward. These aspects will be further discussed in Section 4.1.2.

#### 4.1.1. Deposition Parameters

In this subsection, the selected parameters for experimentation and their results on surface roughness and overall finishing/superficial defects will be evaluated.

The first deposition parameters strategy was not successful in providing a smooth metal transfer. During deposition on the cylinder, wire sticking was observed. The wire feed speed was high for that particular travel speed, which caused the heat input to be insufficient to fully melt the wire.

Therefore, partially melted material was deposited, and bead fusion was inadequate, ultimately interrupting the process. Albeit not having presented good results on finishing and general structure, this first deposition was further used in the validation of the DNN model regarding quality assurance and surface roughness classification, further commented on in Section 4.6 and Section 4.7.

**Figure 5:**
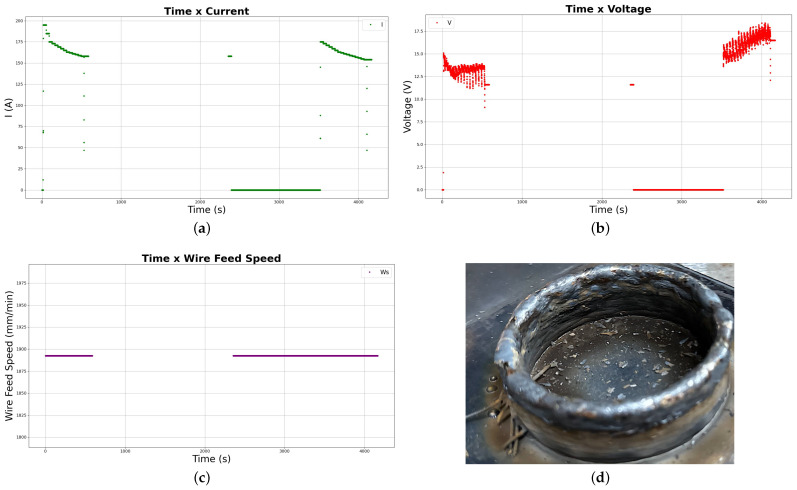
(**a**) Current graph of deposited part using the first strategy, (**b**) voltage graph, (**c**) wire feed speed graph (constant throughout the deposition), and (**d**) picture from the deposited part.

Deposition parameters used in the first test of part deposition are related in Table 2. Those parameters were varied during the deposition and captured by the Digital Twin data pipeline to be stored in Google Cloud. Graphs obtained from the history of data available in the Cloud Firestore database are shown in the grid of images in Figure 5, presenting current, voltage and wire feed speed variation through time.

The cooling time is noticeable in the graphs, which show a difference of around 25 min between the two deposition sessions.

Figure 5d shows the first experimental deposited part. Variation of thickness among layers has been observed, primarily caused by material overflow at the top layers.

The noted superficial defects (major stubbing, overbuilding, balling, and necking), besides the general overbuilding in a “ladder” effect, can be seen in the picture taken from the deposited part.

The first experimental deposited part presented a poor superficial finishing with a great variation of thickness among layers and poor quality overall, needing several improvements for a smoother part with better general quality through the refining of deposition parameters and trajectory strategies.

The second successful experiment resulted in a part of fair overall quality, with modified input deposition parameters when compared to the first experiment, but using the same step-by-step approach with cooling dwell time between layers similar to the first strategy and deposited part. The main difference was the increase in the travel speed from 5 to 7 mm/s, eliminating the wire-sticking problem and therefore the stubbing observed in the first part. Input current was also diminished. All input parameters are presented in Table 3.

**Figure 6:**
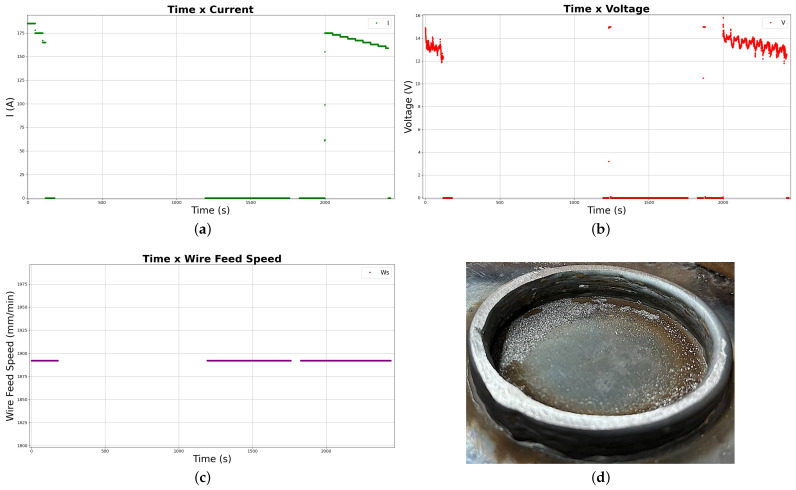
(**a**) Current graph of deposited part using the second strategy, (**b**) voltage graph, (**c**) wire feed speed graph (constant throughout the deposition), and (**d**) a picture of the second deposited part.

The variation of the parameters throughout the deposition can be observed in the graphs in Figure 6, and the better overall finishing with fewer superficial defects (minor necking and the expected ladder effect of lateral overbuilding) when compared to the first experiment can be inferred from the picture of the real deposited part in Figure 6d.

**Figure 7:**
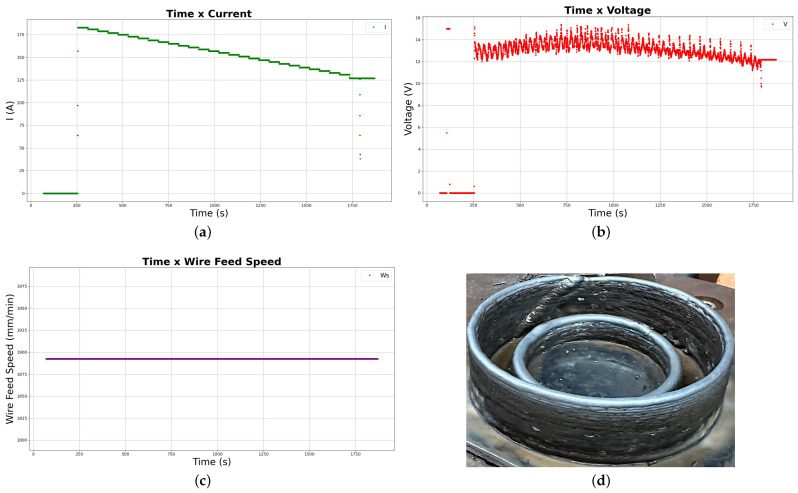
(**a**) Current graph of deposited part using the second strategy, (**b**) voltage graph, (**c**) wire feed speed graph (constant throughout the deposition), and (**d**) picture from the third deposited part (the outer hollow cylinder).

Compared to the first deposition, the second and third successful experiments achieved better results in terms of general surface finish and bead defect reduction, as illustrated in Figure 7d.

The improvement in surface quality stems from parameter selection refinement (by increasing the travel speed as in the previous experiment), which prevents previous wire sticking defects while allowing a higher cooling rate, effectively mitigating the detrimental effects of excessive heat accumulation.

Throughout the third deposition, the pre-programmed *Z*-axis torch increment, calibrated from previous experiments, introduced deviations in the expected arc gap, leading the metal transfer mode to fluctuate depending on the wire’s entry position into the arc. Initially, a continuous bridge transfer mode was observed, whereas in the subsequent layers the transfer became intermittent, a shift associated with reduced layer height and higher molten pool temperature. As the current decreased, both the distance between the deposited layer and the tungsten electrode and the metal transfer mode were restored. This behavior is clearly reflected in the arc voltage versus deposition time shown in Figure 7b.

The parameters used for the third successful experiment are shown in Table 4, while their variation over the deposition time is presented in the grid of graphs in Figure 7.

**Figure 8:**
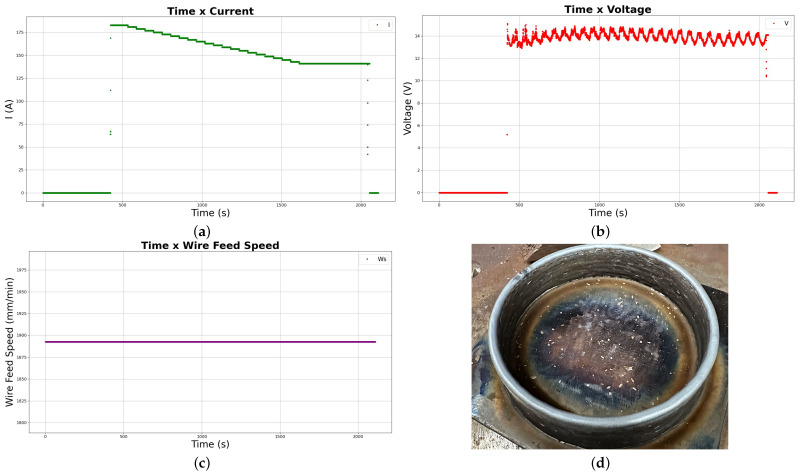
(**a**) Current graph of deposited part using the third strategy, (**b**) voltage graph, (**c**) wire feed speed graph (constant throughout the deposition), and (**d**) picture from the fourth deposited part.

Another strategy that was analyzed through the experimental deposition process was the continuous helical deposition. Overall, the deposition remained stable, characterized by a bridge transfer mode, which can be inferred from the periodicity observed in Figure 8b. Nevertheless, the arc gap increased to 5 mm at the end of the first layer. Thus, a lack of fusion occurred on the substrate, and the wire was unable to spread adequately, leading to localized balling at that point. The strategy parameters are summarized in Table 5.

Having also a lower steep downward variation of the current when compared to the first and second experiments, the variations in the parameters throughout the deposition can be seen in the graphs of the grid in Figure 8.

Finally, the overall good quality of superficial roughness with little noted defects (some overbuilding at the top layer and minor necking) can be inferred from the picture taken of the finished part in Figure 8d.

#### 4.1.2. Trajectory Definition and Metal Deposition Strategies

During extensive deposition tests conducted at the robotic manufacturing cell within the GRACO laboratory, the helical deposition method emerged as the superior strategy along the *Z*-axis. This finding aligns with recent literature, which often highlights the advantages of continuous deposition paths, such as helical or spiral trajectories, over unidirectional (e.g., zigzag) methods in WAAM [43,44]. The helical approach was further optimized by dynamically adjusting the arc current, progressively reducing its value as the wall height increased during the deposition process.

**Figure 9:**
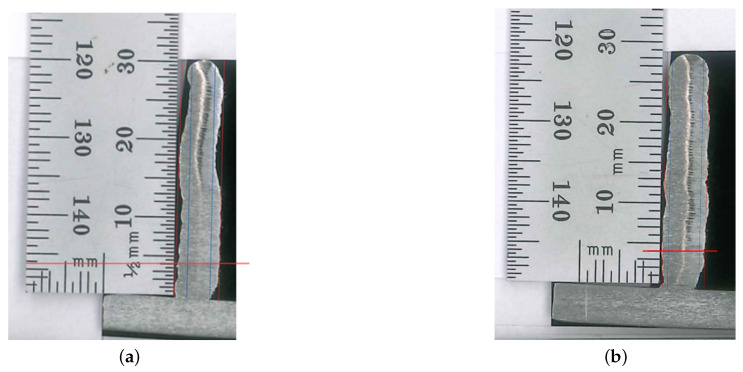
(**a**) Cross-sectional third part, produced by stepwise strategy, and (**b**) cross-sectional fourth part, produced by helical strategy. Blue and red lines in the pictures are to highlight the layer’s effective and total wall width, respectively, used for measurements and comparisons made between the experiments.

Figure 9 presents the cross-sections of the third and fourth parts after the cutting process. For clarity, the red dashed lines demarcate the total wall width, while the blue dashed lines indicate the effective wall width. The solid red line marks the boundary of the fourth deposited layer. Measurements commenced at the fourth layer and proceeded at 1 mm intervals along the entire part height. The results for the third specimen showed an effective wall width of 2.9 mm and a total wall width of 5.4 mm. Alternatively, the fourth specimen measured 3.9 mm and 5.3 mm, respectively. The mean wall thickness was calculated as 5.40 ± 0.07 mm for the third specimen and 5.20 ± 0.05 mm for the fourth. Furthermore, data from the deposition tests revealed a significant difference in surface quality. The fourth specimen, fabricated using a helical deposition strategy, exhibited approximately 43% lower surface waviness, 1.2 mm vs. 0.68 mm (which is given by the amplitude of total and effective width), compared to the third part, despite both being produced with identical parameters up to the 22nd layer.

According to Wang et al. [45], heat continuously accumulates in each deposited layer until a balance is reached between the heat input and dissipation to the previous layers and the environment. Since this heat buildup influences the deposited morphologies and structures, it becomes necessary to adjust the process parameters according to the height. Therefore, a gradual reduction in the current was applied as a strategy to keep the melt pool width within a narrow range, increasing process stability. In this study, the current was the only parameter varied and, in the GTAW process, it acts independently of the wire feed speed, thus being the main factor controlling the deposited layer width. The width increase caused by the rise in interpass temperature was counterbalanced by the reduction resulting from the decrease in current. Since the cross-sectional area of the layer remained constant (the cross-sectional area of the layer is determined by the relationship between the wire feed speed and the travel speed, since these parameters were not varied, it can be concluded that the area remained unchanged), any change in width implied a corresponding adjustment in the layer height, which thus remained within a stable and uniform range.

However, since the proposed QA analysis methods need to be tested and validated in various scenarios, other trajectory strategies are also contemplated through the validation process, such as layer deposition in sessions with pauses for cooling down after a certain number of layers deposited, and non-continuous step-by-step ascent from one layer to another, but without pauses. The first method allows the general thermal gradient of the system (metal support build plate + deposited part) to settle before another session of layer deposition, whilst the second one briefly allows the molten pool to begin solidifying before starting a new layer.

A comparative analysis of the deposition strategies revealed that the stepwise ascending method, both with and without interpass cooling, resulted in inconsistent bead geometry and surface irregularities in the as-built condition, unlike the more uniform outcomes of the continuous ascending helical approach. Also, the step-by-step trajectory definition strategy inherently results in a “ladder” aspect of layering thickness at the point of TCP ascending, which is due to material being fed into the molten pool while the positioning table is not turning, accumulating material into a single point of the part’s wall.

**Figure 10:**
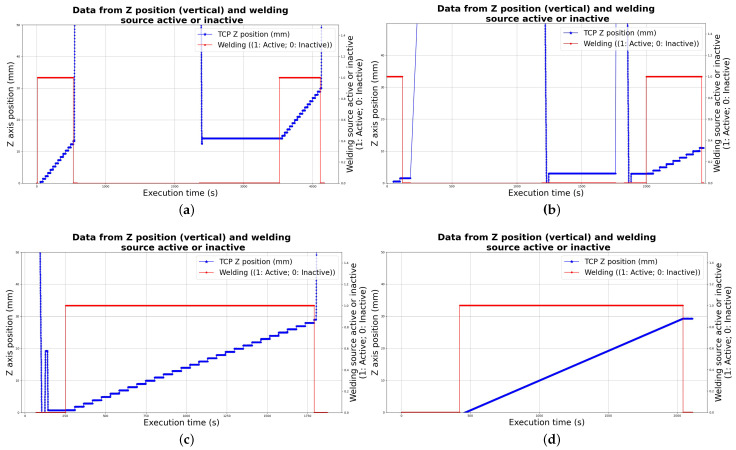
(**a**) *Z* position graph of deposited part using the two-session cooling strategy, (**b**) *Z* position graph of the second experiment, (**c**) *Z* position graph of the second strategy part, and (**d**) *Z* position graph of the helical strategy deposited part.

The variation of the *Z* position throughout the deposition process of the first, second, and third (helical) strategies and experiments can be observed in a grid of graphs obtained via the Cloud Firestore database provided in Figure 10.

The trajectory definition for material deposition has a direct impact on the superficial layering quality in the deposited part’s walls. This is due to the material accumulation on the step-based ascension in *Z* position in the first and second strategies, characterizing an overbuilding defect in the wall. The difference is clearly visible in the provided graphs of *Z* position throughout the deposition time of the defined strategies, which also reinforces the importance of the Digital Twin in supporting decision making in production cycles and refining parameters for the deposition of metal parts in the manufacturing cell.

Another thing to consider is the mentioned cooling time between two sessions of layer deposition, which is easily noticeable in the *Z* position graph (a) in Figure 10. The rough finishing and the easily visible discontinuous bead from the superior half of the deposited part are possibly due to the great thermal inertia and abrupt thermal gradient caused by the difference in temperature of the different halves. Better continuous cooling strategies [46,47] might be considered in the future to address those problems and prevent the observed defects from happening.

Both the helical method and the step-by-step methods presented a beginning of balling and necking effects in the first two layers. However, as the energy input/output and thermal gradient were stabilized as layers were deposited, those defects faded and the bead became continuous for most of the length of the deposited parts’ walls.

**Figure 11:**
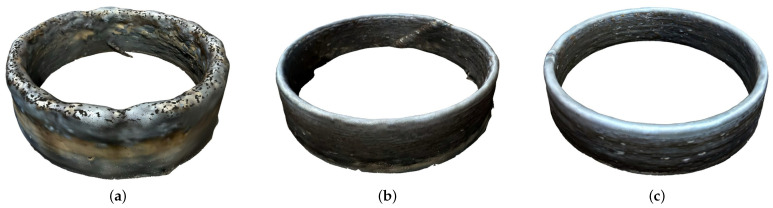
(**a**) Scanned 3D model of the deposited part from the first experiment, (**b**) scanned 3D model of the third experiment’s part, and (**c**) scanned 3D model of the helical strategy experiment.

The comparison between the overall quality obtained on parts deposited with those deposition methods can be inferred by the grid of images in Figure 11. Those images were generated from the 3D models scanned via PolyCam software iPhone app version 4.0.11, using the photogrammetry method of 3D surface generation.

As perceptible in the grid of images, the step-by-step deposition presents a rougher finish when compared to the helical one. Also, the discontinuous height ascent produces excesses of material in specific parts when the tip of the tool is going up by melting more steel in that little time period, when the linear horizontal deposition speed is slowed down for the layer switching process.

**Figure 12:**
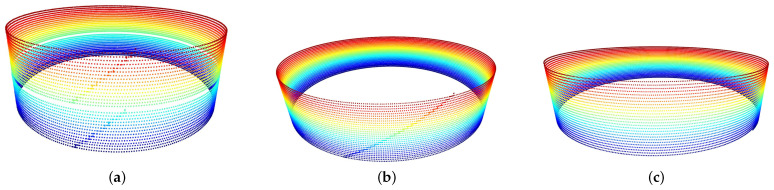
(**a**) Three-dimensional point cloud of the deposited part from the first experiment, (**b**) three-dimensional point cloud of the third experiment’s part, and (**c**) three-dimensional point cloud of the helical strategy experiment. The gradient of colors is used for better contrast and visualization, as well as to highlight the change in height throughout the *Z*-axis layering.

The differences of the layering processes of the three conducted experiments are also perceivable in the form of the captured point clouds from the Digital Twin pipeline. The *X* and *Y* positions are corrected via the stored θ angle of the positioning table to reconstruct the observed point cloud during deposition, also using the *Z* position as the measurement of the height of the deposited layer. The grid of images of those point clouds is presented in Figure 12

In the first experiment in Figure 12, there are two separate point clouds for each deposition section, with the step-by-step layering being clearly visible in each one of them. In the second point cloud, third experiment, there is only a single deposition section, with a step-by-step layering clearly visible through the wall of the captured point cloud, whereas in the third point cloud, fourth experiment, corresponding to the helical strategy, there is a smooth, continuous ascent of the TCP for the layering, resulting in a continuous deposition without accumulated material and captured points in the form of a “ladder” that was observed in the previous experiments.

To validate the alignment quality between the Digital Twin-generated point cloud and the photogrammetry scans, quantitative error metrics were computed across the four specimens (Table 6). Post-convergence, the alignment yielded an average Root Mean Square Error (RMSE) of 2.03 mm (σ=0.58), confirming robust global registration within the expected tolerance of the rough WAAM surface. The Hausdorff distance, representing the maximum local deviation between the planned trajectory and the actual deposited surface, averaged 6.50 mm (with a maximum of 8.39 mm in Experiment 3). This high peak deviation quantitatively captures the amplitude of the significant physical defects—such as localized balling and layer overbuilding—that were subsequently flagged by the classification network.

It is noteworthy that the maximum Hausdorff distance (8.39 mm) reflects a compound of localized physical defects and the global scale drift inherent to markerless photogrammetry. However, this absolute dimensional variance does not impact the downstream surface quality analysis. The PointNet classification pipeline explicitly normalizes all input segments to a unit sphere prior to feature extraction. Moreover, the DT point clouds are not used for the PointNet-based classification, as molten pool width and height are not currently being captured by the DT, being a limitation of the framework, but it serves as a basis for comparison and validation of deposition strategies and trajectories, and for the validation of the DT efficiency on capturing the essential deposition trajectory. Consequently, the model is scale-invariant, relying solely on the relative topological complexity (surface roughness and connectivity) rather than absolute metric dimensions.

### 4.2. Data ETL Pipeline and Modules

Throughout the following sections, all modules that compose the expected pipeline framework already presented in Figure 4 are explained in their own following subsections.

The ETL pipeline is a sub-entity of the Digital Twin Entity in the proposed Digital Twin architecture framework presented in the methodology and illustrated in Figure 2.

### 4.3. Data Extraction

The first part of the pipeline comprises the extraction of data to be transformed and loaded into the local repositories and the cloud infrastructure for the history of variables and analysis results’ documents. The data extraction is a crucial part for the correct functioning of the whole data pipeline; if any errors occur in part scanning or mesh generation, those errors will be propagated through the entirety of the implemented data framework.

Throughout the following sub-subsections, the utilized methods for data extraction and 3D mesh generation methods for comparisons and CAD/CAM deposition trajectory definition will be summarized.

#### 4.3.1. Photogrammetry Scanning 3D Point Cloud Acquisition

The chosen method of 3D scanning, utilizing the iPhone 16 Pro Max’s camera, is the photogrammetry-based scan of the manufactured parts. Having a theoretical spatial resolution of approximately 0.3 mm in photo-based scans, the PolyCam [48] software app version 4.0.11 photogrammetry solution was the chosen image fetch and processing mechanism. While preliminary manual verification estimated a global dimensional scale drift (absolute error in height/diameter) of 1–2 mm due to the markerless scanning approach, this global deviation does not impact the downstream analysis, as the proposed PointNet classification relies on unit-sphere normalized segments.

For the GTAW-based deposited samples of hollow parts, a sampling of 160–180 pictures per deposited part was taken (RAW mode for maximum resolution, macro mode, 20–30 cm of distance from the deposited part) for generating the 3D meshes in the PolyCam iPhone app. This dataset size, which is approximately 3 to 4 times the standard recommendation for this geometry [48], ensures high overlap (>80%) to mitigate occlusion and filter transient specular reflections during 3D mesh generation. The complete dataset, including source images and photogrammetry parameters (distance from referential, camera orientation, blur score), is available in [49].

Acquired 3D meshes from the photogrammetry method applied to hollow metal parts are used for validation of the PointNet surface roughness classification model described in Section 4.6.

#### 4.3.2. Digital Twin-Based Positional Data 3D Point Cloud Acquisition

Since the original Digital Twin data pipeline culminates in storing near-real-time acquired data, if the robotic cell’s sensors are correctly calibrated and the trajectory is well defined based on the reference CAD models for deposition, the Digital Twin-acquired positional data is then a good source of comparison for geometrical features of the deposited parts.

Among the variables acquired from the robotic AM cell, positional data, deposition parameters, linear TCP speed, positioning table rotating angle, and TCP orientation are all parameters that can be used to precisely reconstruct the trajectory of the deposition process. All this collected information can then be used to support AI-based analytical models applied to the data related to each deposition experiment.

All the 3D models of deposited hollow parts described in the data extraction subsection must be corrected and prepared before being analyzed by the proposed AI model. The transformation processes, corrections, and overall data structuring of the ETL pipeline are detailed in the following subsection.

### 4.4. Data Transformation

Comprising the main part of the ETL pipeline framework, the data transformation part is composed of many modules of the flowchart and is divided into many programs that must be executed in a specific sequence in order to properly handle and prepare the data for the analytical algorithms and the proposed adapted DNN model.

#### 4.4.1. SPSR Density Correction and Superficial Normal Generation

After the referential correction and noise filtering of 3D meshes, one final step to guarantee the homogeneity of 3D point clouds to be analyzed in future steps is to apply the Superficial Poisson Superficial Reconstruction [50] to correct any inhomogeneities present in the point distribution along the surface of the point clouds.

This method is applied to correct any uneven scanning of manufactured 3D parts, reducing the occurrence of high densities in some point graph neighborhoods, as opposed to other point graph neighborhoods with almost no scanned points.

SPSR was selected to homogenize point density across the surface, a critical preprocessing step to ensure consistent input for the PointNet architecture. While SPSR induces a smoothing effect, empirical observation confirmed that it preserves the meso-scale geometric deviations relevant to the defined quality classes, while effectively filtering high-frequency noise from the photogrammetry process. Alternative methods, such as the Ball Pivoting Algorithm (BPA), were evaluated but rejected due to the generation of non-manifold geometry. The target density was standardized to 49,152 points per model. This resolution was chosen to align with the neural network’s architecture, allowing for the extraction of exactly 24 segments of 2048 points. Empirical tests showed that doubling the resolution to 98,304 points significantly increased computational load on mesh processing and training times without necessarily improving classification accuracy.

After the density correction of the point clouds, the immediate vertex and mesh normals are computed using the corresponding methods on the open3D library. Then, new triangle meshes are generated based on the corrected points and calculated normals.

In the following sub-subsections, the last steps of data transformation for load and further analysis are commented on, including the segmentation of the transformed point clouds and vectorization/compression for being compatible with AI models’ input parameterization.

#### 4.4.2. Point Cloud Segmentation

A crucial part of the data pre-processing performed before inputting it to the classification DNN model is the segmentation of acquired data. This part of the pipeline is directly responsible for how the analytical models “see” the manufactured parts, thus being vital for the computer vision part of the dataflow. In fact, any modifications in this process, as small as they might be, have profound impacts on the weights of feature inputs and activation of neural nodes, contributing even further to the unpredictability of the classification models’ learning process, training results, and even validation.

After some iterations of experimental training and testing of the adapted PointNet model, it was decided to use a radial split of the 3D models along the *Z* axis. This was made to uniformize the segments of the point clouds, making it easier to normalize input ranges and to reduce distortions in the edges of the segments. Another advantage of this method of segmentation is the possibility of evaluating continuity among deposited layers.

Since the input segments are made of 2048 points in the point cloud each, and the SPSR sampling of the 3D point clouds is set at 49,152 points for each STL input, the radial angle of slicing throughout the *Z* axis is set at 15º. ICP correction is then applied to the segments in order to minimize the mean distances among the point clouds with respect to the reference frame, so those discrepancies do not become a bias when the segments are fed to the neural network surface roughness classification model. This alignment is critical to eliminate positional biases—such as variations in radial distance or angular offset—that would skew the subsequent unit-sphere normalization. The algorithm was configured with a distance threshold of 0.02 using a Point-to-Point estimation method. Since the initial orientation is constrained by the radial segmentation logic, the alignment is robust, ensuring that the neural network processes purely topological features rather than positional artifacts.

After the segmentation is finished, the classification of the real parts’ segments is performed using an unsupervised learning approach based on the Fiedler number, a spectral graph metric that quantifies surface connectivity. For each 3D point cloud segment, a k-nearest neighbors (KNN) graph is constructed and its normalized Laplacian is computed to extract the second-smallest eigenvalue (Fiedler number) [51], as calculated from the formula in Equation (1). These values serve as input features for clustering via the K-means algorithm, which groups segments into predefined quality classes. Segments are then assigned to quality classes (i.e., good, fair, poor) based on their cluster’s mean Fiedler value, and the results are stored and summarized for further analysis.

**Figure 13:**
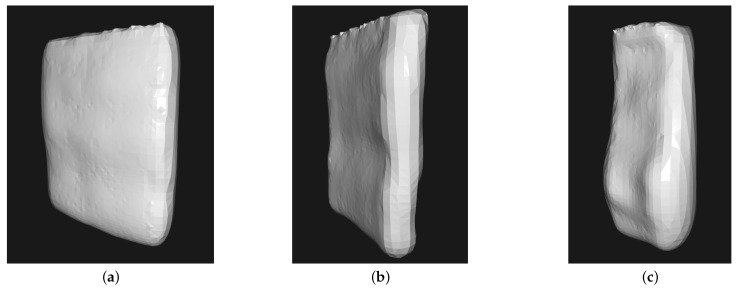
(**a**) Example of a segment classified as “Good” quality, (**b**) “Fair” quality segment example, and (**c**) “Poor” quality segment example.

The classification results of all the segments obtained via the Fiedler number-based KNN clusters’ classification are presented in Table 7. Also, examples from classified segments from each quality level can be observed in Figure 13.

(1) $$\lambda_{2}={\mathrm{eig}}_{2}\left(L\right),\;\;L=I-D^{-1/2}AD^{-1/2}$$

where:

- $\lambda_{2}$—Fiedler number, the second-smallest eigenvalue of the normalized Laplacian L;
- ${\mathrm{eig}}_{2}\left(\cdot \right)$—function that returns the second-smallest eigenvalue of a matrix;
- L—normalized Laplacian matrix of the graph;
- *I*—identity matrix of size n×n, where *n* is the number of nodes in the graph;
- *A*—adjacency matrix of the k-nearest neighbors graph constructed from the point cloud;
- *D*—degree matrix, a diagonal matrix where $D_{ii}=\sum_{j}A_{ij}$.

#### 4.4.3. Data Vectorization and Compression

After the segmentation process, the data is not yet ready to be fed into the DNN model’s input. Being the last step before the application of the PointNet model, the data vectorization and compression convert the input segments into structured tensors containing positional coordinates, surface normals, and class labels.

For training and testing sets, the pipeline applies randomized data augmentation to increase sample diversity and improve model generalization. Augmentation includes rotation (primarily around the *Z*-axis), scaling, and Gaussian jittering, with augmentation factors tailored to each class to balance representation. Augmentation factors for each class, along with the intervals of parameters for augmentation on each technique, are presented in Table 8.

Each segment is resampled to a fixed number of points (e.g., 2048 points) and normalized to fit within a unit sphere, ensuring consistency across samples. The final output is stored in compressed HDF5 format, enabling efficient loading and training within the deep learning pipeline.

#### 4.4.4. Quality Assurance Analysis Model

Finally, the last data transformation process relates to the analysis model used to classify manufactured parts and compare the 3D models. Those QA assessment sessions use the transformed, segmented, and vectorized 3D models’ data as inputs, having collections of classes and feature comparison results as outputs.

The real parts’ photogrammetry-based 3D point clouds are also processed through the entire transformation pipeline in order to make them ready for the training process of an AI model. The analytical AI model used is based on Deep Neural Networks (DNNs) adapted from PointNet and will be further discussed in Section 4.6.

### 4.5. Data Load

Three-dimensional models that have their origin referential and density corrected are transformed into triangle-based .STL meshes to be stored in local collections.

Then, after the referential correction, segmentation, and vectorization (in the case of transformed .csv data), the resulting vectorized point clouds are then analyzed, and the resulting classification documents after the quality classification performed by the neural network model are capable of being stored in the Google Cloud Firestore Database.

### 4.6. PointNet for 3D Point Cloud Superficial Roughness Classification

The selection of a Deep Neural Network architecture was driven by the limitations of classical machine learning approaches in this specific domain. Preliminary investigations utilizing baseline classifiers (SVM, Random Forest) trained on manually extracted geometric features—such as height variance, mean thickness, and centroid distance—proved insufficient, failing to generalize across the dataset. These global statistical features could not adequately resolve the subtle, localized topological defects (meso-scale roughness) inherent to the WAAM process. Consequently, PointNet [42] was selected specifically for its capability to process raw, unordered point sets directly. This allows the model to learn complex, non-linear spatial features through shared Multi-Layer Perceptrons (MLPs) without the information loss associated with manual feature engineering.

**Figure 14:**
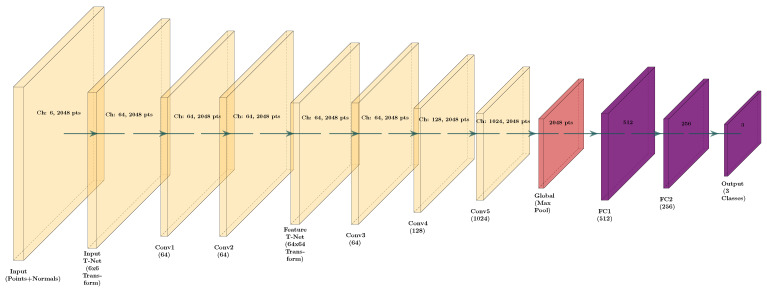
Adapted PointNet classification network simplified model architecture (adapted from [42]).

The DNN model for classification was created based on the PointNet classification model [42], adapting the model for TensorFlow 2 and altering the shape of the input nodes to be compatible with normals + positional data input, using vectors of 6 positions to include the normal vector for each point instead of the original positional points, with only 3 positions input. The simplified architecture and layer visualization of the adapted PointNet model can be seen in Figure 14.

Input 3D parts were segmented into 2048-point point clouds compatible with the neural network input nodes. To enhance the model’s geometric awareness, each input point cloud is normalized to fit within a unit sphere, and its associated surface normals are normalized to unit length. These two components—spatial coordinates and normals—are concatenated into a six-dimensional tensor, which is then passed through an input transformation network that learns to align the data spatially. The transformed input undergoes a series of shared multi-layer perceptrons (MLPs), implemented as 1D convolutional layers, to extract local features from each point. A second transformation network refines the feature space by enforcing invariance to geometric distortions, and the resulting features are aggregated into a global descriptor using max pooling.

The global feature vector is passed through a series of fully connected layers, each incorporating batch normalization and dropout to enhance generalization and reduce overfitting. This sequence culminates in a final classification layer that produces logits—raw, unnormalized scores representing the model’s confidence for each of the three surface quality classes. These logits are later transformed into probabilities using activation functions such as softmax. To mitigate class imbalance and support stable training, the model leverages a focal loss function with conservative parameters, which places greater emphasis on challenging examples that are harder to classify. Furthermore, a regularization term is applied to the transformation matrices to promote orthogonality and maintain geometric consistency. Together, these components enable the model to effectively learn both local and global spatial features, making it well-suited for assessing surface roughness in metal additive manufacturing parts.

### 4.7. Analysis Results

In this subsection, the analytical results of the application of the PointNet-based model of superficial roughness classification as the baseline for quality assurance of manufactured parts are presented. Testing and validation of those are presented in the following sub-subsections. Training evaluation metrics are also explained in the following sub-subsections, which are then further used to evaluate the effectiveness and overall learning generalization of the model.

#### 4.7.1. Evaluation Criteria

To assess the performance of the seat part detection system in a production setting, five key metrics are considered: Accuracy, Precision, Recall, mean Average Precision (mAP), and F1-score. These indicators help evaluate the system’s ability to detect defects reliably and consistently.

#### 4.7.2. Accuracy

Accuracy measures the overall correctness of the model’s predictions. It represents the proportion of correctly classified instances—both defective and non-defective—relative to the total number of samples.

(2) $$\mathrm{Accuracy}=\frac{TP+TN}{TP+TN+FP+FN}$$

where:

- TP: True Positives—defective parts correctly identified.
- TN: True Negatives—non-defective parts correctly identified.
- FP: False Positives—non-defective parts incorrectly flagged as defective.
- FN: False Negatives—defective parts missed by the model.

#### 4.7.3. Precision

Precision evaluates the reliability of positive predictions. It measures the proportion of correctly identified defective parts among all parts classified as defective.

(3) $$\mathrm{Precision}=\frac{TP}{TP+FP}$$

where:

- TP: True Positives.
- FP: False Positives.

#### 4.7.4. Recall

Recall, also known as Sensitivity or True Positive Rate, measures the proportion of actual defective parts that were successfully detected.

(4) $$\mathrm{Recall}=\frac{TP}{TP+FN}$$

where:

- FN: False Negatives.

#### 4.7.5. Mean Average Precision (mAP)

mAP is commonly used in object detection tasks. It calculates the average precision across all classes and thresholds, providing a comprehensive view of the model’s detection performance.

(5) $$\mathrm{mAP}=\frac{1}{N}\sum_{i=1}^{N}AP_{i}$$

where:

- $AP_{i}$: Average Precision for class *i*.
- *N*: Total number of classes.

#### 4.7.6. F1-Score

The F1-score combines Precision and Recall into a single metric by calculating their harmonic mean. It is especially useful when the dataset is imbalanced.

(6) $$\mathrm{F1-score}=\frac{2\cdot \mathrm{Precision}\cdot \mathrm{Recall}}{\mathrm{Precision}+\mathrm{Recall}}$$

This score balances the trade-off between Precision and Recall, offering a more nuanced view of the model’s effectiveness.

#### 4.7.7. Confidence Interval

To evaluate the statistical robustness of the reported performance metrics, specifically the overall accuracy, a 95% Confidence Interval (CI) is calculated. This metric quantifies the uncertainty associated with the point estimate, providing a probabilistic range within which the true model performance is expected to lie. Given the fixed size of the testing dataset, the Normal Approximation method (Wald Interval) is applied:

(7) $$\mathrm{CI}=\hat{p}\pm Z\times \sqrt{\frac{\hat{p}\left(1-\hat{p}\right)}{N}}$$

where:

- $\hat{p}$: The observed performance metric (e.g., overall accuracy).
- *Z*: The Z-score corresponding to the desired confidence level (1.96 for a 95% confidence level).
- *N*: The total number of samples in the testing dataset.

This interval estimates the uncertainty around the observed accuracy $\hat{p}$ by giving a 95% range of plausible values for the true accuracy that accounts for sampling variability in the test set.

#### 4.7.8. Training and Testing on Parts’ Superficial Roughness Classification

The training of the adapted PointNet surface roughness classification model was performed on real segments of parts that were treated with the described ETL pipeline, using mixed segments from all four real parts deposited, described in Section 4.1, splitting into 70% train and 20% test datasets with data augmentation, and a third balanced dataset for post-validation with 10% of the segments being used without data augmentation for post-training separate evaluation based solely on mixed segments of the real deposited parts. The hyperparameters that resulted in the best overall generalization of the adapted PointNet model and accuracy are presented in Table 9.

Training routines were rejected/accepted according to their generalization and overall balanced accuracy throughout the 3 classes. Training routines and epochs that resulted in a balanced overall accuracy of over 65% were separated for further validation on the separate validation dataset.

**Figure 15:**
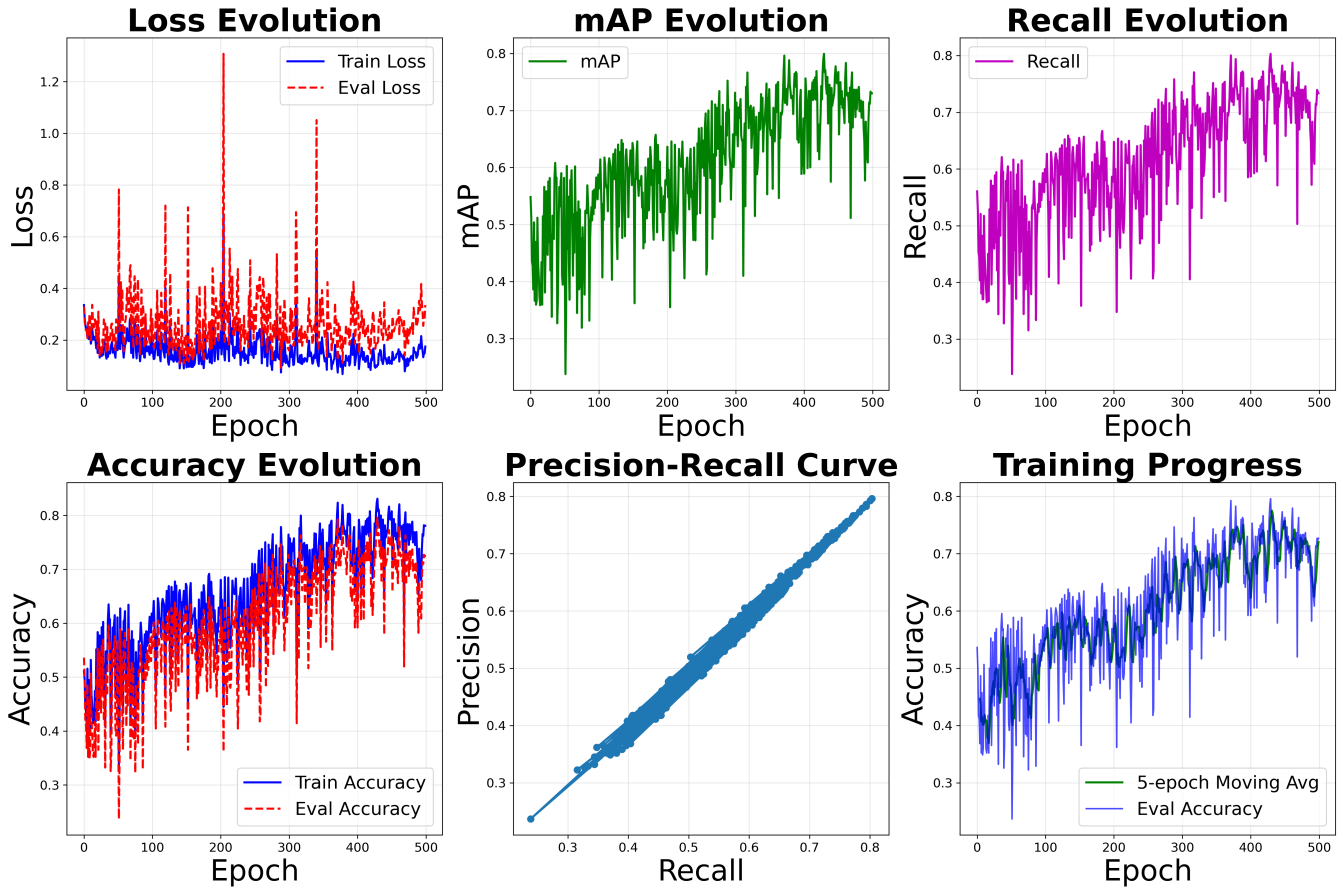
Training metrics from the adapted PointNet model on the mixed data-augmented random segments from all the experiments of the real deposited parts.

Metric evaluation graphs were generated from training of the adapted PointNet model throughout 500 epochs, with the best overall model on evaluation being achieved at epoch 402. Said graphs can be observed in Figure 15.

The training analysis of the model demonstrates a consistent and well-behaved learning process across multiple performance metrics. The loss evolution graph shows a gradual decline in both training and evaluation loss, indicating effective convergence without signs of overfitting. The mAP and recall curves steadily rise over epochs, reflecting improved precision and sensitivity in classification. Accuracy metrics for both training and evaluation follow an upward trajectory, with evaluation accuracy slightly trailing—a healthy sign of generalization. The precision–recall scatter plot reveals a balanced trade-off, with points clustering along the diagonal, while the training progress graph confirms stable learning through a rising epoch average and evaluation accuracy. Together, these visualizations validate the robustness of the model and its suitability for surface roughness classification tasks in metal additive manufacturing.

However, volatility in the loss, along with noticeable noise across all other metrics (mAP, Recall, and Accuracy), indicates an unstable training run, potentially due to a high diversity of data with a small sample size of similar segments with similar superficial roughness characteristics. This instability makes it difficult to reliably assess the model’s true performance at any single epoch, but the general evaluation shows an evolution in general accuracy.

#### 4.7.9. Testing and Validation Results

**Figure 16:**
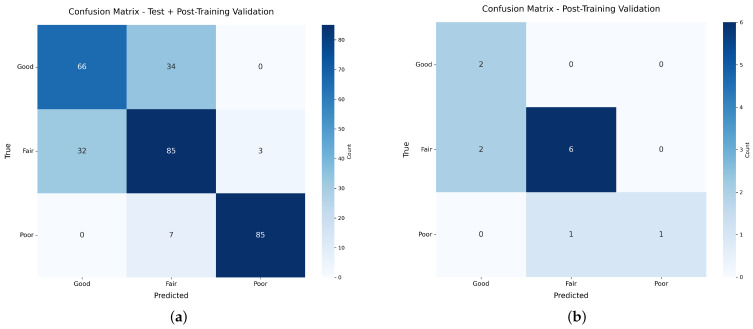
(**a**) Confusion matrix of the application of the adapted PointNet model on Test + Post-training Validation segments’ data. (**b**) Confusion matrix on Post-training Validation segments only.

Results from testing and post-training unaugmented segments’ data validation of the PointNet-based model are presented in Table 10, with their respective confusion matrices presented in Figure 16.

The PointNet-based neural network has a fair accuracy of 75.64%, keeping in mind that the three classes that serve as input have very strict tolerances for different classifications among input classes, with high variability on input data, which contrasts with the small dataset available when considering the very difficult task of evaluating surface roughness quality of real manufactured parts using 3D point clouds. The achieved accuracy is considered substantial, particularly when acknowledging that PointNet’s original function was 3D shape classification. The shift to surface roughness demands a higher sensitivity to localized superficial details, making this quality assessment task considerably more challenging.

## 5. Discussion

While the model’s overall accuracy is 75.64%, its utility in the manufacturing environment is underscored by its robust performance in identifying the most critical failures. Specifically, analysis of the testing results table and confusion matrix (Table 10(a) and Figure 16a) reveals a high-confidence detection for the Poor quality class, achieving a Precision of 0.97 and Recall of 0.92. This reliable capability to isolate unacceptable parts is the key requirement for industrial quality control, enabling automated decision making, such as process shutdown or flagging parts for immediate rework, which is crucial for reducing material waste and optimizing production cycles.

Regarding the statistical robustness of the 75.64% accuracy, a 95% confidence interval was calculated based on the combined evaluation sample size (*N* = 322), yielding a margin of error of ±4.69%. While the overall accuracy reflects the challenge of distinguishing between the subtle topological boundaries of “Good” and “Fair” classes using only geometric data, the model demonstrates exceptional performance where it matters most for Quality Assurance: defect detection. The classification report (Table 10) indicates a Recall of 0.92 and Precision of 0.97 for the “Poor” class. This confirms that while the model may occasionally misclassify acceptable surface variations, it is highly reliable at identifying significant deviations, minimizing the risk of false negatives (Type II errors) in an industrial inspection context.

The framework for QA analysis on surface roughness classification for metal AM parts was proven successful in helping with the assessment of process parameter tuning when combined with the data acquired from the Digital Twin dataflow. The applied methodology helped in manufacturing better-quality parts, progressing from each experiment.

The classification of positional data 3D models captured from the Digital Twin and via images contributes to fault detection over sent commands and stored data when compared to the real results of the part obtained. Imperfections concentrated in specific areas of the real part might contribute to parameter tuning—such as current, wire feed speed, and height offset from the molten pool—when analyzed with the data stored via the DT. Scanning and classifying those 3D models of real parts also helps in having a labeled history of data, further used to improve the deposition process.

When compared to quality inspection AI models and machine learning applied on manufacturing processes [52,53], similar results are found, with evaluation accuracies ranging from 80 to 99 percent. Another example of the application of Deep Neural Networks on the quality inspection of parts is found in Alvares et al. [24], where a defect classification system for LMD-wire printed parts is proposed using YOLOv5s and Faster R-CNN architectures, having achieved an average detection accuracy of 0.932 and 0.872, respectively. The models were trained to detect five defect classes (Balling, Dripping, Necking, Overbuilding, Stubbing) across four quality levels. Different from the pipeline implemented in this current work, those applications do not use DT data for comparisons, and 3D point cloud surface evaluation of metal AM parts is not commonly found in the state of the art of metal AM QA analysis.

Like every analysis framework, the one proposed in this work has its own scope limitations. Since only tubular hollow parts were tested, it is expected that for better results on parts of other shapes and materials, there must be training on a higher variety of segments from diverse methods of deposition and topologies of parts. Limiting the scope to hollow parts, it is expected to train the model on hollow cubes, slopes, and pyramid trunks in the future to test the Fiedler number-based surface roughness classification framework and to explore different backbones for the deep neural network structure.

Also, in the specific topic of ground truth labelling, a distinction must be drawn regarding the definition of “roughness” in this framework. The classification pipeline relies on spectral graph connectivity metrics (Fiedler number) to generate ground truth labels. This metric quantifies topological complexity and geometric irregularity (meso-scale roughness), such as bead waviness and layer step-over artifacts. While standard Quality Assurance often relies on $R_{a}$ or $R_{z}$ metrics, preliminary investigations indicated that such metrics were unreliable for the acquired dataset due to the resolution limits of the photogrammetry pipeline.

The necessary application of SPSR functions as a low-pass filter, effectively smoothing out the high-frequency micro-peaks required for accurate $R_{a}$ calculation while preserving the global geometric continuity. Consequently, attempting to map the generated 3D segments to standard ISO roughness values would be methodologically unsound without higher-resolution metrology. Instead, the classification relies on the Fiedler Number as a mathematically objective proxy for topological complexity, robustly quantifying the significant geometric deviations relevant to WAAM process stability. It is observed that physical defects manifest as distinct quantitative shifts in the spectral domain. For instance, ‘necking’ defects act as bottlenecks in the k-nearest neighbors graph, mathematically reducing the algebraic connectivity (Fiedler number), while ‘balling’ introduces localized density variations. This spectral quantification allows the framework to objectively categorize defects based on their topological severity rather than subjective visual interpretation. Therefore, the integration of laser profilometry for micro-feature correlation is proposed as a specific avenue for future work.

Additionally, future research will specifically address the rigorous metrological validation of the photogrammetry pipeline. While the current macro-mode capture provides sufficient local resolution (≈0.3 mm) for the proposed topological classification, quantifying the global scale drift (preliminarily estimated at 1–2 mm) against calibrated references (such as Coordinate Measuring Machines) is necessary to extend the framework from qualitative defect detection to precise dimensional tolerance inspection.

The proposed methodology has its specific focus on quantifying surface roughness via topological connectivity. The Fiedler number-based approach excels at evaluating the general continuity of the 3D surface graph but is not designed to identify or classify other significant defects that are primarily visual in nature. Superficial anomalies such as discoloration, minor porosity, or spatter, which may not substantially alter the 3D graph connectivity, are therefore not captured by this analysis. These types of defects are often better suited for 2D image-based inspection methods. For example, approaches using Convolutional Neural Networks (CNNs) like YOLO can be trained to detect and classify a wide range of specific visual anomalies from photographs of the deposited part, as demonstrated in related work on defect classification [24]. The current pipeline should therefore be seen as a complement to, rather than a replacement for, these image-based QA systems.

Another limitation is the model’s inability to evaluate key deposition efficiency metrics, such as dimensional accuracy or material usage. This is an intentional design choice, as the radial segmentation process, along with vectorization, normalization and compression, was developed to mitigate the influence of general geometric properties, like the part’s overall diameter or curvature, in order to isolate the surface roughness texture. As a result, the pipeline in its current form cannot be used to measure the effective deposited wall thickness or the precise cross-sectional area of each layer. This “deposition efficiency” is a crucial metric in WAAM for analyzing process stability, calculating operational costs, and predicting material consumption. Furthermore, the photogrammetry scanning method, while excellent for external surfaces, does not capture the internal cross-sectional geometry, which is where these efficiency metrics are most accurately and usually measured [54].

Furthermore, the framework was validated based on the Cold Wire Gas Tungsten Arc Welding process; however, as a generalized framework for surface roughness evaluation of metal additively manufactured parts, it is expected to validate it on other process-based metal manufactured parts, such as DED LMD-wire and the more common GMAW process.

All the algorithms, datasets, and the pipeline described throughout this article can be found in an authorial online repository available in https://github.com/MASCAM/PointNet_Superficial_Roughness_Classifier_Hollow_Metal_AM_Parts (accessed on 20 October 2025). Separate data for photogrammetry parameters and pictures of the real deposited parts can be found in [49].

## 6. Conclusions

In this work, an implementation of a QA pipeline based on a Digital Twin for additive manufacturing was presented. The proposed pipeline consists of the acquisition of positional data via the presented Digital Twin pipeline to generate point clouds or meshes generated through images using PolyCam 3D. Those meshes are then aligned via the ICP method and have their density corrected via the Screened Poisson Surface Reconstruction method. After such corrections, the point clouds are segmented and classified according to a k-nearest neighbors graph constructed from the calculated Fiedler number of the segments to serve as input for training an adapted DNN model based on PointNet.

The adapted PointNet neural network model achieved an accuracy of 75.64% in classifying surface roughness from 3D scans of real metal deposited parts. This result demonstrates the feasibility of the proposed framework to validate the deposition process and support the tuning of Digital Twin parameters. While the current iteration highlights limitations in evaluating contiguous low-roughness zones, the successful classification of unaugmented validation segments confirms that the model generalizes to new geometric data within the tested domain. This establishes a robust methodological foundation for intelligent QA in metal AM, shifting the focus from simple visual inspection to deep geometric topology analysis.

Moreover, the use of PointNet in robotic GTAW AM cells aligns with the broader trend of intelligent manufacturing, where AI-driven models enhance automation and precision. By leveraging Digital Twin simulations, manufacturers can optimize deposition strategies based on historical data, ensuring consistent surface quality across different builds. This approach can minimize post-processing requirements, such as grinding or polishing, which are typically needed to correct surface irregularities. The ability to classify roughness at surface level also supports quality assurance protocols, ensuring compliance with industry standards.

A significant evolution of the current project would be to transition the framework from a post-process quality assurance (QA) tool to a near real-time process monitoring and control system. This extension would involve integrating an in situ vision sensor, such as a camera equipped with a UV filter, mounted directly on the robotic manufacturing cell. While the current pipeline relies on commanded positional data from the Digital Twin (DT) and post-process photogrammetry scans, this new approach would capture the actual deposition geometry during the process. As demonstrated in studies on vision-based sensing for arc welding [55], such a camera setup can effectively filter the intense arc glare and allow for the real-time measurement of key bead characteristics. This would provide a high-frequency stream of data on the true molten pool width and deposited layer height, which would be stored in the DT to create a much higher-fidelity representation of the as-built part.

## Figures and Tables

**Table 1:** Bibliometric comparison of DT-based works in manufacturing.

Article	No. Citations	Proposal or Implementation	Objective of Analysis	Monitored Process	Uses DT	Standard or Architecture	Protocols Used	Database Type	Analysis Model
[5]	303	DT model for rotating machinery in manufacturing	Simulation for fault diagnosis of rotor systems	Subtractive manufacturing	Yes	Own	N/A	Simulated data	Heuristic
[6]	325	Simulation of supplier selection in digital manufacturing	Risk mitigation in supply chain management	General	No	N/A	N/A	Simulated	KNN, logistic regression, supervised ML
[7]	295	DT for production line design	Optimize production efficiency	Production line	Yes	N/A	OPC	Local	Heuristic
[8]	155	Digital Twin for cutting machine	DT for subtractive manufacturing based on LISA architecture	Subtractive manufacturing	Yes	LISA, ISO 13399 [9]	Enterprise Service Bus (ESB)	Local	ML proposed
[10]	9	Use of point cloud for manufactured parts	SGT applied to dimensional integrity analysis	Additive manufacturing	No	N/A	N/A	N/A	Heuristic, SGT, Fiedler
[11]	24	Scan of 3D printed parts for surface analysis	Align parts with 3D models to detect defects	Additive manufacturing	No	N/A	N/A	N/A	Heuristic
[12]	312	DT for AM with Blockchain	Blockchain-based DT for aerospace	Additive manufacturing	Yes	N/A	Blockchain protocol (DIGEST)	Distributed Blockchain	N/A
[13]	326	DT with statistical analysis for glass process	Improve efficiency and speed of glass manufacturing	Glass production line	Yes	SCADA	OPC, Ethernet	Cloud	Heuristic
[14]	291	Deep transfer learning for fault analysis	Fault analysis	Assembly line	Yes	Own architecture (DFDD)	N/A	Local PVS	Deep learning, transfer learning, DFDD
[15]	20	3D printing mesh from scan	Create prototypes of cultural artifacts	Additive manufacturing	No	N/A	N/A	N/A	Heuristic, PCAM param.
Current work	N/A	DL for mesh and failure analysis	Improve efficiency and quality in AM	Additive manufacturing	Yes	ISO 23247 [16]	MQTT, HTTP, Ethernet	Firebase (Cloud)	DL and Fuzzy

Abbreviations: Art. = Article, Cit. = Citations, Prop. = Proposal, Impl. = Implementation, Std. = Standard, Arch. = Architecture, Prot. = Protocols, DB = Database, mfg. = manufacturing, mgmt. = management, LR = logistic regression, ML = machine learning, ESB = Enterprise Service Bus, SGT = Spherical Geometry Theory, DL = deep learning, AM = additive manufacturing, DT = Digital Twin, OPC = OLE for Process Control, MQTT = Message Queuing Telemetry Transport, HTTP = Hypertext Transfer Protocol, PCAM = Point Cloud Analysis Method, DFDD = Deep Fault Detection and Diagnosis, PVS = Production Validation System, N/A = Not Applicable.

**Table 2:** GTAW Parameters and dimensional aspects used in the first experiment using the first strategy mentioned (top) and deposition dimensional parameters (bottom).

GTAW Deposition Process Setup Overview (First Experiment, Step-by-Step Strategy)	
Arc Current (A) first half	195 → 158 (firstly 2 steps of −10 A then steps of −2 A and steps of −1 A at the second half of the first session)
Arc Current (A) second half	175 → 154 (steps of −2 A and steps of −1 A at the second half of the second session)
Arc Voltage (V) first half	Approx. 11.00–15.19
Arc Voltage (V) second half	Approx. 13.50–18.50
Wire Feed Speed (mm/min)	1893 (constant)
Travel Speed (mm/s)	5 (constant)
Trajectory strategy	Step-by-step approach with cooling time
**Dimensional Parameters**	
Mean height (mm)	28.5
Number of layers	30
Mean layer height (mm)	0.95
Mean total thickness (mm)	6.7
Programmed deposition diameter (mm)	80
Passes per layer	1

**Table 3:** GTAW parameters used in the second experiment using the second strategy mentioned (top) and deposition dimensional parameters (bottom).

GTAW Deposition Process Setup Overview (Second Experiment, Step-by-Step Strategy)	
Arc Current (A) first half	185 → 165 (2 steps of −10)
Arc Current (A) second half	175 → 159 (steps of −2 A)
Arc Voltage (V) first half	Approx. 14.88–11.86
Arc Voltage (V) second half	Approx. 15.80–11.78
Wire Feed Speed (mm/min)	1893 (constant)
Travel Speed (mm/s)	7 (constant)
Trajectory strategy	Step-by-step approach with cooling time
**Dimensional Parameters**	
Mean height (mm)	12.4
Number of layers	12
Mean layer height (mm)	1.033
Mean total thickness (mm)	6.5
Programmed deposition diameter (mm)	80
Passes per layer	1

**Table 4:** GTAW parameters used in the third experiment using the second strategy mentioned (top) and deposition dimensional parameters (bottom).

GTAW Deposition Process Setup Overview (Third Experiment, Second Step-by-Step Strategy)	
Arc Current (A)	183 → 127 (steps of −2 A)
Arc Voltage (V)	Approx. 11.14–15.42
Wire Feed Speed (mm/min)	1893 (constant)
Travel Speed (mm/s)	7 (constant)
Trajectory strategy	Step-by-step approach without cooling time
**Dimensional Measurements**	
Mean effective height (mm)	30.9
Number of layers	29
Mean layer height (mm)	1.065
Mean total thickness (mm)	5.4
Programmed deposition diameter (mm)	120
Passes per layer	1

**Table 5:** GTAW parameters used in the fourth experiment using the helical *Z* position ascension strategy (top) and deposition dimensional parameters (bottom).

GTAW Deposition Process Setup Overview (Fourth Experiment, Helical Strategy)	
Arc Current (A)	183 → 141 (steps of −2 A)
Arc Voltage (V)	Approx. 12.84–15.14
Wire Feed Speed (mm/min)	1893 (constant)
Travel Speed (mm/s)	7 (constant)
Trajectory strategy	Helical approach
**Dimensional Parameters**	
Mean height (mm)	29.4
Number of layers	29
Mean layer height (mm)	1.014
Mean total thickness (mm)	5.2
Programmed deposition diameter (mm)	120
Passes per layer	1

**Table 6:** Quantitative alignment metrics between Digital Twin-generated point clouds and photogrammetry scans post-ICP registration for comparisons.

Experiment	Fitness	RMSE (mm)	Hausdorff Dist. (mm)
1	1.00	1.76	7.54
2	1.00	1.39	4.49
3	1.00	2.97	8.39
4	1.00	1.98	5.60
**Mean ± Std. Dev.**	**1.00 ± 0.00**	**2.03 ± 0.58**	**6.50 ± 1.54**

**Table 7:** Segment classification summary based on Fiedler Number surface connectivity metric.

Experiment	Total Segments	Good	Fair	Poor	Fiedler Mean
First Experiment	24	3 (12.5%)	19 (79.2%)	2 (8.3%)	0.002386
Second Experiment	24	3 (12.5%)	14 (58.3%)	7 (29.2%)	0.007046
Third Experiment	24	6 (25.0%)	18 (75.0%)	0 (0.0%)	0.001359
Fourth Experiment	24	4 (16.7%)	20 (83.3%)	0 (0.0%)	0.000752
Overall	96	16 (16.7%)	71 (74.0%)	9 (9.4%)	0.002886

**Table 8:** Data augmentation factors and augmentation technique parameters for 3-class point cloud classification.

Class	Class Name	Augmentation Factor	
Class 0	Good	36	
Class 1	Fair	8	
Class 2	Poor	90	
**Total training segments:** 1328. **Test:** 310.			
Augmentation Technique	Parameter	Value	Application Probability
Rotation (*Z*-axis)	Angle Range	±30°	80%
Rotation (*X*/*Y*-axis)	Angle Range	±10°	30% (if *Z*-axis applied)
Scaling	Scale Factor Range	0.9–1.1	60%
Jittering	Standard Deviation	0.01	70%

**Table 9:** Training hyperparameters for 3-class adapted PointNet model.

Hyperparameter	Value	Description
**Training**		
Batch Size	16	Samples per batch
Max Epochs	500	Maximum training epochs
Learning Rate (Initial)	0.0005	Base learning rate
Learning Rate (Minimum)	0.000001	Minimum learning rate
Decay Step	20,000	Steps for learning rate decay
Decay Rate	0.95	Learning rate decay factor
Minimum Class Accuracy	0.65	Threshold for model saving
**Optimizer (Adam)**		
Beta1	0.9	First moment decay rate
Beta2	0.999	Second moment decay rate
Epsilon	$1\times 10^{-8}$	Numerical stability term

**Table 10:** (**a**) Evaluation metrics of the adapted PointNet model on Test + Post-training Validation segments’ data. (**b**) Evaluation metrics on Post-Training Validation segments only.

(a)	
	PointNet—Test + Post-training validation metrics
Accuracy	0.7564
F1-Score per Class	
Good	0.67
Fair	0.69
Poor	0.94
Classification Report	
Good	Precision: 0.67, Recall: 0.66
Fair	Precision: 0.67, Recall: 0.71
Poor	Precision: 0.97, Recall: 0.92
(**b**)	
	PointNet—Post-training validation metrics
Accuracy	0.7500
F1-Score per Class	
Good	0.67
Fair	0.80
Poor	0.67
Classification Report	
Good	Precision: 0.50, Recall: 1.00
Fair	Precision: 0.86, Recall: 0.75
Poor	Precision: 1.00, Recall: 0.50

## Data Availability

All data generated throughout this study, along with instructions on how to use it and all used algorithms, can be found at https://github.com/MASCAM/PointNet_Superficial_Roughness_Classifier_Hollow_Metal_AM_Parts (accessed on 20 October 2025).

## Abbreviations

The following abbreviations are used in this manuscript:

ABB	ASEA Brown Boveri
AI	Artificial Intelligence
AM	Additive Manufacturing
CAD	Computer-Aided Design
CAM	Computer-Aided Manufacturing
CNC	Computer Numerical Control
CPS	Cyber-Physical System
CW-GTAW	Cold Wire Gas Tungsten Arc Welding
DBSCAN	Density-Based Spatial Clustering of Applications with Noise
DCDCE	Data Collection and Device Control Entity
DED	Direct Energy Deposition
DT	Digital Twin
DTUE	Digital Twin User Entity
DNN	Deep Neural Network
ETL	Extraction, Transformation, Load
FDM	Fused Deposition Modeling
FFF	Fused Filament Fabrication
HMI	Human–Machine Interface
ICP	Iterative Closest Point
IoT	Internet of Things
LMD	Laser Metal Deposition
LiDAR	Light Detection and Ranging
ML	Machine Learning
MQTT	Message Queuing Telemetry Transport
RA-CNN	Residual-Attention Convolutional Neural Network
SGT	Spectral Graph Theory
SLS-SLM	Selective Laser Sintering—Selective Laser Melting
SPSR	Screened Poisson Surface Reconstruction
SVM	Support Vector Machine
TCP	Tool Center Point
TS	Torch Speed
WAAM	Wire Arc Additive Manufacturing
WFS	Wire Feed Speed

## References

[1] Dutta Pramanik P., Mukherjee B., Pal S., Upadhyaya B., Dutta S. (2019). Ubiquitous Manufacturing in the Age of Industry 4.0: A State-of-the-Art Primer. A Roadmap to Industry 4.0: Smart Production, Sharp Business and Sustainable Development. Advances in Science, Technology & Innovation.

[2] Santos B., Charrua-Santos F.M.B., Lima T.M. Industry 4.0: An Overview, 2018. https://api.semanticscholar.org/CorpusID:211545607.

[3] Bong Kim D., Shao G., Jo G. (2022). A digital twin implementation architecture for wire + arc additive manufacturing based on ISO 23247. Manuf. Lett..

[4] Lee J., Park H.J., Chai S., Kim G.R., Yong H., Bae S.J., Kwon D. (2021). Review on Quality Control Methods in Metal Additive Manufacturing. Appl. Sci..

[5] Wang J., Ye L., Gao R., Li C., Zhang L. (2019). Digital Twin for rotating machinery fault diagnosis in smart manufacturing. Int. J. Prod. Res..

[6] Cavalcante I., Frazzon E., Forcellini F., Ivanov D. (2019). A supervised machine learning approach to data-driven simulation of resilient supplier selection in digital manufacturing. Int. J. Inf. Manag..

[7] Liu Q., Zhang H., Leng J., Chen X. (2018). Digital twin-driven rapid individualised designing of automated flow-shop manufacturing system. Int. J. Prod. Res..

[8] Botkina D., Hedlind M., Olsson B., Henser J., Lundholm T. (2018). Digital Twin of a Cutting Tool. Procedia CIRP.

[9] (2006). Cutting Tool Data Representation and Exchange—Part 1: Overview, Fundamental Principles and General Information Model.

[10] Rao P., Kong Z., Duty C., Smith R., Kunc V., Love L. (2015). Assessment of Dimensional Integrity and Spatial Defect Localization in Additive Manufacturing (AM) using Spectral Graph Theory (SGT). J. Manuf. Sci. Eng..

[11] Decker N., Wang Y., Huang Q. (2020). Efficiently registering scan point clouds of 3D printed parts for shape accuracy assessment and modeling. J. Manuf. Syst..

[12] Mandolla C., Petruzzelli A., Percoco G., Urbinati A. (2019). Building a Digital Twin for Additive Manufacturing through the Exploitation of Blockchain: A case analysis of the aircraft industry. Comput. Ind..

[13] Zhang H., Liu Q., Chen X., Zhang D., Leng J. (2017). A Digital Twin-Based Approach for Designing and Multi-Objective Optimization of Hollow Glass Production Line. IEEE Access.

[14] Xu Y., Sun Y., Liu X., Zheng Y. (2019). A Digital-Twin-Assisted Fault Diagnosis Using Deep Transfer Learning. IEEE Access.

[15] Tashi, Ullah A.S., Watanabe M., Kubo A. (2018). Analytical Point-Cloud Based Geometric Modeling for Additive Manufacturing and Its Application to Cultural Heritage Preservation. Appl. Sci..

[16] (2021). Automation Systems and Integration—Digital Twin Framework for Manufacturing—Part 1: Overview and General Principles.

[17] Newcombe R., Izadi S., Hilliges O., Molyneaux D., Kim D., Davison A., Kohli P., Shotton J., Hodges S., Fitzgibbon A. KinectFusion: Real-Time Dense Surface Mapping and Tracking. Proceedings of the 2011 10th IEEE International Symposium on Mixed and Augmented Reality, ISMAR 2011.

[18] Tomar B., Shiva S. (2023). Cold metal transfer-based wire arc additive manufacturing. J. Braz. Soc. Mech. Sci. Eng..

[19] Singh R., Singh R. (2020). 3—Welding and joining processes. Applied Welding Engineering.

[20] Singh A., Negi S., Kapil S., Karunakaran K., Das M. (2021). A comprehensive study of auxiliary arrangements for attaining omnidirectionality in additive manufacturing machine tools. J. Manuf. Sci. Eng..

[21] Syed W.U.H., Li L. (2005). Effects of wire feeding direction and location in multiple layer diode laser direct metal deposition. Appl. Surf. Sci..

[22] Costello S.C., Cunningham C.R., Xu F., Shokrani A., Dhokia V., Newman S.T. (2023). The state-of-the-art of wire arc directed energy deposition (WA-DED) as an additive manufacturing process for large metallic component manufacture. Int. J. Comput. Integr. Manuf..

[23] Liu M., Kumar A., Bukkapatnam S., Kuttolamadom M. (2021). A Review of the Anomalies in Directed Energy Deposition (DED) Processes & Potential Solutions—Part Quality & Defects. Procedia Manuf..

[24] Alvares A.J., Betancourth B.S.F., Cabral J.V.A., Lacroix I. (2025). Automated defect classification in additive manufacturing LMD-wire using deep learning. J. Braz. Soc. Mech. Sci. Eng..

[25] Liu C., Le Roux L., Körner C., Tabaste O., Lacan F., Bigot S. (2022). Digital Twin-enabled Collaborative Data Management for Metal Additive Manufacturing Systems. J. Manuf. Syst..

[26] Charrier Q., Hakam N., Benfriha K., Meyrueis V., Liotard C., Bouzid A.H., Aoussat A. (2023). Towards the Augmentation of Digital Twin Performance. Sensors.

[27] Doungtap S., Petchhan J., Phanichraksaphong V., Wang J.H. (2023). Towards Digital Twins of 3D Reconstructed Apparel Models with an End-to-End Mobile Visualization. Appl. Sci..

[28] Rao P., Kong Z., Duty C., Smith R. (2016). Three Dimensional Point Cloud Measurement Based Dimensional Integrity Assessment for Additive Manufactured Parts Using Spectral Graph Theory. International Manufacturing Science and Engineering Conference.

[29] Cabral J.V.A., Álvares A.J., de Carvalho G.C. (2024). Digital Twin Implementation for an Additive Manufacturing Robotic Cell based on the ISO 23247 Standard. IEEE Lat. Am. Trans..

[30] Cabral J.V.A., da Cunha Facciolli A.C., de Carvalho G.C., Álvares A.J. (2026). Digital Twin-Enabled Quality Assurance Analysis of Metal Manufactured Parts Based on Neural Networks Applied to 3D Meshes. Flexible Automation and Intelligent Manufacturing: The Future of Automation and Manufacturing: Intelligence, Agility, and Sustainability, Proceedings of FAIM 2025, New York City, NY, USA, 21–24 June 2025.

[31] Jin L., Zhai X., Kang W., Zhang K., Wu D., Nazir A., Jiang J., Liao W.H. (2024). Big data, machine learning, and digital twin assisted additive manufacturing: A review. Mater. Des..

[32] Bellini C., Berto F., Cocco V.D., Iacoviello F., Mocanu L.P., Razavi N. (2021). Additive manufacturing processes for metals and effects of defects on mechanical strength: A review. Procedia Struct. Integr..

[33] Wu W., Xue J., Xu W., Lin H., Tang H., Yao P. (2021). Parameters Optimization of Auxiliary Gas Process for Double-Wire SS316L Stainless Steel Arc Additive Manufacturing. Metals.

[34] Hou M., Li P., Cheng S., Yv J. (2024). CNN-based defect detection in manufacturing. Adv. Control Appl..

[35] Fountas N.A., Kechagias J.D., Vaxevanidis N.M. (2023). Optimization of Selective Laser Sintering/Melting Operations by Using a Virus-Evolutionary Genetic Algorithm. Machines.

[36] Mohamed O.A., Masood S.H., Bhowmik J.L. (2021). Modeling, analysis, and optimization of dimensional accuracy of FDM-fabricated parts using definitive screening design and deep learning feedforward artificial neural network. Adv. Manuf..

[37] Li X., Jia X., Yang Q., Lee J. (2020). Quality analysis in metal additive manufacturing with deep learning. J. Intell. Manuf..

[38] Ester M., Kriegel H.P., Sander J., Xu X. A Density-Based Algorithm for Discovering Clusters in Large Spatial Databases with Noise. Proceedings of the Knowledge Discovery and Data Mining.

[39] Kazhdan M.M., Hoppe H. (2013). Screened poisson surface reconstruction. ACM Trans. Graph..

[40] Open3D Team (2018). Open3D: A Modern Library for 3D Data Processing. arXiv.

[41] Jain K. (2020). How Photogrammetric Software Works: A Perspective Based on UAV’s Exterior Orientation Parameters. J. Indian Soc. Remote Sens..

[42] Qi C.R., Su H., Mo K., Guibas L.J. (2016). PointNet: Deep Learning on Point Sets for 3D Classification and Segmentation. arXiv.

[43] Venturini G., Montevecchi F., Bandini F., Scippa A., Campatelli G. (2018). Feature based three axes computer aided manufacturing software for wire arc additive manufacturing dedicated to thin walled components. Addit. Manuf..

[44] Sarma R., Kapil S., Joshi S.N. (2025). A comparative analysis of trochoidal toolpath with traditional toolpaths used in wire arc-based directed energy deposition process. Prog. Addit. Manuf..

[45] Wang H., Kovacevic R. (2001). Rapid prototyping based on variable polarity gas tungsten arc welding for a 5356 aluminium alloy. Proc. Inst. Mech. Eng. Part B J. Eng. Manuf..

[46] Wang H., Cao L., Li Y., Schneider M., Detemple E., Eggeler G. (2021). Effect of cooling rate on the microstructure and mechanical properties of a low-carbon low-alloyed steel. J. Mater. Sci..

[47] Reisgen U., Sharma R., Mann S., Oster L. (2020). Increasing the manufacturing efficiency of WAAM by advanced cooling strategies. Weld. World.

[48] PolyCam Polyform—A 3D Scanning and Reconstruction Software, 2025. https://poly.cam/.

[49] Cabral J.V.A. PointNet_Superficial_Roughness_Classifier_Hollow_Metal_AM_Parts, 2025. https://ieee-dataport.org/documents/pointnetsuperficialroughnessclassifierhollowmetalamparts.

[50] Kazhdan M.M., Bolitho M., Hoppe H. Poisson surface reconstruction. Proceedings of the Eurographics Symposium on Geometry Processing.

[51] Wang C., Samari B., Siddiqi K. (2018). Local Spectral Graph Convolution for Point Set Feature Learning. arXiv.

[52] Rydzi S., Zahradnikova B., Sutova Z., Ravas M., Hornacek D., Tanuska P. (2024). A predictive quality inspection framework for the manufacturing process in the context of industry 4.0. Sensors.

[53] Sankhye S., Hu G. (2020). Machine learning methods for quality prediction in production. Logistics.

[54] Aldalur E., Veiga F., Suárez A., Bilbao J., Lamikiz A. (2020). Analysis of the Wall Geometry with Different Strategies for High Deposition Wire Arc Additive Manufacturing of Mild Steel. Metals.

[55] Xiong J., Pi Y., Chen H. (2019). Deposition height detection and feature point extraction in robotic GTA-based additive manufacturing using passive vision sensing. Robot. Comput.-Integr. Manuf..

